# Species boundaries in the messy middle—A genome‐scale validation of species delimitation in a recently diverged lineage of coastal fog desert lichen fungi

**DOI:** 10.1002/ece3.8467

**Published:** 2021-12-19

**Authors:** Jesse Jorna, Jackson B. Linde, Peter C. Searle, Abigail C. Jackson, Mary‐Elise Nielsen, Madeleine S. Nate, Natalie A. Saxton, Felix Grewe, María de los Angeles Herrera‐Campos, Richard W. Spjut, Huini Wu, Brian Ho, H. Thorsten Lumbsch, Steven D. Leavitt

**Affiliations:** ^1^ Department of Biology Brigham Young University Provo Utah USA; ^2^ Science & Education The Grainger Bioinformatics Center The Field Museum Chicago Illinois USA; ^3^ Departamento de Botánica Instituto de Biología Universidad Nacional Autónoma de México Ciudad De México Mexico; ^4^ World Botanical Associates Bakersfield California USA; ^5^ Monte L. Bean Life Science Museum Brigham Young University Provo Utah USA

**Keywords:** Baja California, Bayes factor delimitation (BFD*), genealogical divergence index (*gdi*), RADseq, species delimitation

## Abstract

Species delimitation among closely related species is challenging because traditional phenotype‐based approaches, for example, using morphology, ecological, or chemical characteristics, may not coincide with natural groupings. With the advent of high‐throughput sequencing, it has become increasingly cost‐effective to acquire genome‐scale data which can resolve previously ambiguous species boundaries. As the availability of genome‐scale data has increased, numerous species delimitation analyses, such as BPP and SNAPP+Bayes factor delimitation (BFD*), have been developed to delimit species boundaries. However, even empirical molecular species delimitation approaches can be biased by confounding evolutionary factors, for example, hybridization/introgression and incomplete lineage sorting, and computational limitations. Here, we investigate species boundaries and the potential for micro‐endemism in a lineage of lichen‐forming fungi, *Niebla* Rundel & Bowler, in the family Ramalinaceae by analyzing single‐locus and genome‐scale data consisting of (a) single‐locus species delimitation analysis using ASAP, (b) maximum likelihood‐based phylogenetic tree inference, (c) genome‐scale species delimitation models, e.g., BPP and SNAPP+BFD, and (d) species validation using the genealogical divergence index (*gdi*). We specifically use these methods to cross‐validate results between genome‐scale and single‐locus datasets, differently sampled subsets of genomic data and to control for population‐level genetic divergence. Our species delimitation models tend to support more speciose groupings that were inconsistent with traditional taxonomy, supporting a hypothesis of micro‐endemism, which may include morphologically cryptic species. However, the models did not converge on robust, consistent species delimitations. While the results of our analysis are somewhat ambiguous in terms of species boundaries, they provide a valuable perspective on how to use these empirical species delimitation methods in a nonmodel system. This study thus highlights the challenges inherent in delimiting species, particularly in groups such as *Niebla*, with complex, relatively recent phylogeographic histories.

## Graphical abstract

We investigate species boundaries and the potential for micro‐endemism for a charismatic genus of lichen fungi occurring in Baja California. Genome‐scale data support high species‐level diversity, along with high levels of endemism, while highlighting the problem of delimiting species, particularly in groups with complex, recent phylogeographic histories.

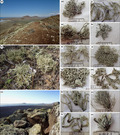

## 1 INTRODUCTION

Species delimitation has never been a simple or straightforward task, due, in part, to how species are conceptualized (Mayden, 1997; Wilkins, 2011). However, conceptualizing species as “separately evolving metapopulation lineages”—the unified species concept (USC)—shifts the debate away from species concepts to how species boundaries can be empirically delimited (de Queiroz, 2007). Under the USC, rather than weighing which property, such as morphology, monophyly, or reproductive isolation, is indicative of a population representing an independent lineage, these properties simply become lines of evidence that can be used within an empirical framework for delimiting species (Sites & Marshall, 2004). Different properties may arise at different times or in different orders during the process of speciation (de Queiroz, 1998, 2005, 2007). Thus, early in the process of speciation, there is often a gray area where species boundaries are difficult to ascertain and potential for conflicting signals among independent lines of evidence (Carstens et al., 2013; Huang & Knowles, 2015; Mallet, 2008). Hence, one of the biggest challenges of species delimitation is in determining when differences in intraspecific population structure become species‐level divergence.

With closely related or recently diverged species, delimitation of species boundaries using traditional phenotype‐based approaches, for example, morphological, ecological, or chemical characteristics, becomes challenging (Poelstra et al., 2020; Printzen, 2010; Wagner et al., 2013). The inclusion of molecular sequence data can be a critical tool for inferring robust species boundaries, especially within an integrative framework (Dayrat, 2005; Fujita et al., 2012; Will et al., 2005). Particularly, high‐throughput sequencing technologies allow for genome‐scale data to be generated using a wide range of time‐ and cost‐effective strategies, potentially generating hundreds to thousands of loci (Hale et al., 2020; McKain et al., 2018). However, even when using genome‐scale data and empirical species delimitation analyses, species boundaries can remain blurred, especially in recently diverged species, hybrid zones, cases of introgression, and when species boundaries remain semipermeable (Harrison & Larson, 2014; Hey et al., 2003; Leaché et al., 2018). Furthermore, harnessing genome‐scale data creates computational challenges and divergent approaches for selecting appropriate genomic loci (Faircloth et al., 2012; Kapli et al., 2020). While additional loci allow for the detection of subtle differences in population structure, potentially resolving recent species‐level divergence (Huang, 2020), these data may also lead to oversplitting populations and inappropriately recognizing populations as species (Rannala, 2015; Sukumaran & Knowles, 2017).

Similar to other nonmodel organismal groups, interpreting species‐level diversity in lichen‐forming fungi has been revolutionized by incorporating DNA sequence data (Lumbsch & Leavitt, 2011; Printzen, 2010; Schneider et al., 2016). Interpreting anatomical, chemical, and morphological characters of lichens within a phylogenetic framework has transformed our understanding of the role of phenotypic characters used for lichen‐forming fungal taxonomy (Printzen, 2010). While portions of the ribosomal cistron, including the internal transcribed spacer (ITS)—the standard barcoding marker for fungi (Schoch et al., 2012), and regions of the mitochondrial genome, have been the standard genetic loci for lichen fungi systematics, genomic data have become increasingly useful when studying closely related species or recently diverged populations (Allen et al., 2018; Alonso‐García et al., 2021; Grewe et al., 2017; Leavitt et al., 2016; Widhelm et al., 2021).

**FIGURE 1:**
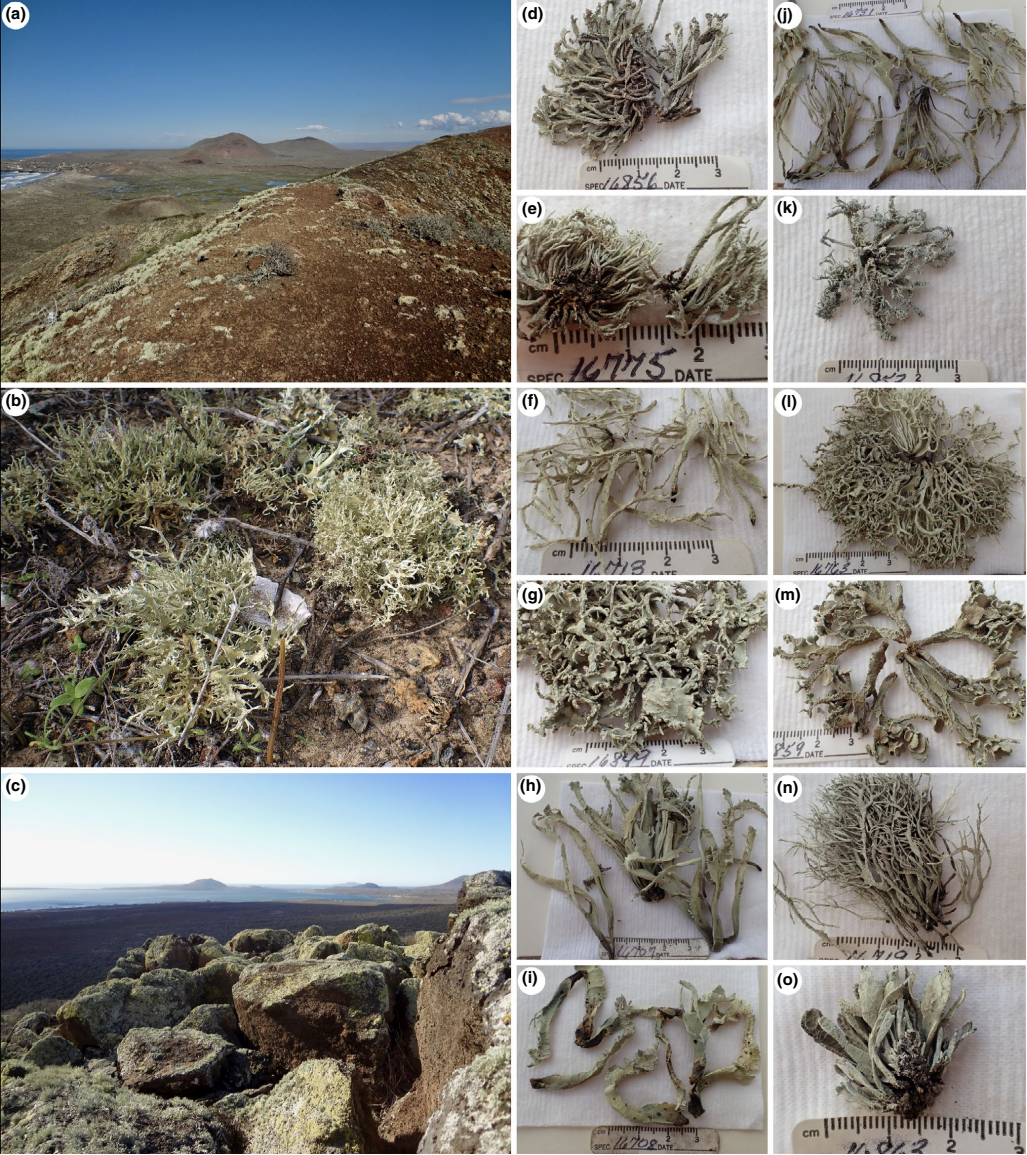
Typic coastal fog desert community in Punta Mazo Nature Reserve, near San Quintín, Baja California, supporting diverse *Niebla* communities. (a) *Niebla* communities on volcanic slopes on Volcán Sudoeste, Punta Mazo. (b) Soil‐dwelling *Niebla arenaria* near Bahía Falsa, San Quintín. (c) *Niebla* communities on West‐facing slopes of Monte Ceniza, Punta Mazo. (d) *Niebla flagelliforma* (*Leavitt 16856BF*). (e) *Niebla* aff. *isidiosa*; this specimen was identified as *N. *aff. *isidiaescens;* it differs by the articulated thallus branches with coralloid isidia whereas *N.* *isidiosa* in panel “k” differs by the rigid thallus with frim branch tips curved downward and isidia occurring moslty along reticulated cortical ridges (*Leavitt 16775*), which closely resembles the type from Isla Guadalpe. (f) *Niebla flabellata* (*Leavitt 16713*). (g) *Niebla* “sp. nov”. (*Leavitt 16849BF*). (h) *Niebla juncosa* var. *juncosa* (*Leavitt 16707*). (i) *Niebla undulata* (*Leavitt 16708*). (j) *Niebla flagelliforma* (*Leavitt 16731*). (k) *Niebla* aff. *isidiosa*; (*Leavitt 16853*). (l) *Niebla marinii* (*Leavitt 16763*). (m) *Niebla lobulata* (*Leavitt 16859BF*). (n) *Niebla juncosa* var. *spinulifera* (*Leavitt 16719*). (o) *Niebla cornea* (*Leavitt 16863BF*). All specimens identified by R. Spjut

Here, we investigate species boundaries in a lineage of fruticose lichen‐forming fungi endemic to coastal fog deserts along the Pacific coasts of the New World—*Niebla* Rundel & Bowler in the family Ramalinaceae. Members of this genus form conspicuous, fruticose lichens, expressing an impressive range of morphological and chemical diversity (Figure 1). This phenotypic variation has resulted in two widely different interpretations of species‐level diversity, either a few, morphologically polymorphic species (Bowler & Marsh, 2004) or a much finer subdivision, two genera, *Niebla* with 42 species and one variety, and *Vermilacinia* with 28 species (Spjut, 1996). These genera were recently investigated within a multilocus, phylogenetic framework (Spjut et al., 2020). *Niebla* diversified more recently than *Vermilacinia*, estimated at ca. 13 Mya, with speciation largely impacted by the climatic oscillations since the Miocene (Spjut et al., 2020). While evidence from Spjut et al. (2020) supports high levels of diversity in *Niebla*, most phenotypically circumscribed *Niebla* species were not recovered as monophyletic using a multilocus dataset. These results have been explained by a hypothesis of micro‐endemism, where populations of unique, allopatric species are found in extremely small distributions within a geographic area. Under a scenario of microendemism, distinct lineages may be masked under morphologically similar forms, even across relatively small geographic areas. Specifically, convergent phenotypes from various, isolated mini‐populations within the coastal fog deserts of California, Baja California, and Baja California Sur would be the norm (Spjut et al., 2020—e.g., *N. homalea*, *N. undulata*; Figure 7). Furthermore, despite generating a robust, multilocus dataset and implementing multiple DNA‐based empirical species delimitation analyses, species boundaries remain uncertain due to discrepancies among different analytical approaches (Carstens et al., 2013; Spjut et al., 2020).

To help resolve the incongruence between the phenotype‐based taxonomy and results from multilocus species delimitation analyses, here we aim to explore the utility of genome‐scale data to resolve species boundaries in this fascinating lineage of lichen‐forming fungi by cross‐validating methodological approaches. Specifically, we used restriction site‐associated DNA sequencing (RADseq) to generate tens of thousands of short loci distributed across the genome to infer evolutionary relationships. Single‐locus approaches using the fungal ITS barcode, as well as several genome‐scale species delimitation analyses, including BPP and SNAPP+Bayes factor delimitation (BFD*), were used to infer species boundaries. BPP is a Bayesian MCMC program for analyzing genomic sequence data under the multispecies coalescent model and has been shown to be able to delimit species boundaries even among closely related lineages (Flouri et al., 2018; Yang & Rannala, 2010, 2014; Zhang et al., 2011). Given that genomic data can be effective in delimiting fine‐scale population structure, rather than species, we use the recently developed heuristic genealogical divergence index (*gdi*) to distinguish between population structure and species boundaries (Jackson et al., 2017; Leaché et al., 2018). With BPP’s estimates of coalescent node heights (*τ*) and ancestral effective population size (*θ*), *gdi* scores can be calculated to estimate the divergence of two taxa (Leaché et al., 2018; Poelstra et al., 2020). Complementarily, we used SNAPP+BFD to rank different species delimitation models. SNAPP attempts to decrease computation time for large amounts of phylogenetic datasets by bypassing the need for gene trees, and species tree estimates are directly inferred from biallelic markers, such as SNP data (Bryant et al., 2012). For each model, Bayes factor scores are calculated based on their marginal likelihood values using a path‐sampling analysis and subsequently compared (Leaché et al., 2014). While this comparison is useful in identifying the best‐fitting species delimitation models, it is computationally limited to datasets with fewer than 10 species and under 100 individual samples (Leaché et al., 2014).

We provide a comparison of species delimitations based on single‐locus data‐the standard DNA barcode for fungi‐with a combination of genome‐scale approaches consisting of: (a) total‐evidence maximum likelihood‐based phylogenetic tree inference, (b) species delimitation using BPP and SNAPP+BFD, and (c) validation of species delimitation models using the genealogical divergence index (*gdi*). Our results provide crucial insights into the diversification history of *Niebla* and highlight the power and limitations of using genome‐scale data to infer species boundaries in recently diverged lineages.

## 2 MATERIAL AND METHODS

### 2.1 Specimen sampling

To investigate evolutionary relationships and the potential for micro‐endemism in *Niebla* species occurring in coastal fog deserts in North America, 182 *Niebla* specimens were collected from Baja California Norte, Mexico in December 2016. Secondary metabolites were identified from all specimens using thin‐layer chromatography (TLC) and following standard methods with solvent systems “C” and “G” (Culberson, 1972; Orange et al., 2001). Specimens were determined by R. Spjut according to his classification (Spjut, 1996). Three additional *Niebla* specimens from the Southern Islands (San Clemente and Santa Barbara Islands), USA, were also included and determined following Spjut (1996). From the initial collections, 40 specimens representing 22 phenotypically circumscribed *Niebla* species and one variety were selected for restriction site‐associated DNA sequencing (RADSeq) to infer evolutionary relationships and delimit species boundaries in this group (Dryad file T1). When possible, multiple representatives of each species collected from different sampling localities were selected. Seven specimens representing *Vermilicinia*, recently shown to be sister to *Niebla*, were used as the outgroup (Spjut et al., 2020). We note that specimens were selected and sequenced before a broad molecular phylogeny was available for *Niebla* (Spjut et al., 2020). A list of all specimens used can be found in the supplementary information (Dryad file T1).

We attempted to amplify the fungal internal transcribed spacer region (ITS), the standard DNA barcoding marker for fungi (Schoch et al., 2012), for the *Niebla* specimens selected for RADSeq. From these specimens, a small portion of the thallus free of visible contamination was excised, and total genomic DNA was extracted using the E.Z.N.A. Plant DNA DS Mini Kit (Omega Bio‐Tek), following the manufacturers’ recommendations. Extractions of specimens for RADseq were done using ZR Fungal/Bacterial DNA MiniPrep Kit (Zymo Research, Irvine, CA, USA) as previously described (Grewe et al., 2017). We amplified the ITS region (ITS1, 5.8S & ITS2) using primers ITS1 (Gardes & Bruns, 1993) with ITS4 (White et al., 1990). Polymerase chain reaction (PCR) amplifications were performed using Ready‐To‐Go PCR Beads (GE Healthcare, Pittsburgh, PA, United States), with cycling parameters following a 66–56°C touchdown reaction (Lindblom & Ekman, 2006). PCR products were visualized on 1% agarose gel and enzymatically cleaned using ExoSAP‐IT Express (USB, Cleveland, OH, United States). Complementary strands were sequenced using the same primers used for amplifications, and sequencing reactions were performed using BigDye 3.1 (Applied Biosystems, Foster City, CA, United States). Products were run on an ABI 3730 automated sequencer (Applied Biosystems) at the DNA Sequencing Center at Brigham Young University, Provo, UT, United States.

### 2.2 Reference sequencing and assembly

We first deep‐sequenced and assembled a reference draft genome from a specimen representing *N. homalea* (Ach.) Rundel & Bowler for identifying mycobiont loci by mapping during the processing of metagenomic RADseq data (*Niebla* culture #47: USA, California, Sonoma Co., Sonoma Coast State Park, Shell Beach, 38.4225, −123.114, on large rock, 25 July 2018, Coll: MH Huhndorf). DNA from this specimen was isolated using the ZR Fungal/Bacterial DNA MiniPrep Kit (Zymo Research, Irvine, CA, USA), converted into libraries with the KAPA Hyper Prep Kit (KAPA Biosciennces, Wilmington, MA, USA) and sequenced at the University of Illinois at Chicago Research Resource Center on Illumina's NextSeq platform as before for lichen‐forming fungi (Grewe et al., 2018). High molecular weight DNA isolation, long‐read sequencing on a Nanopore GridIONx5 sequencer, and assembly of *N. homalea* was done as described before for the lichen fungal culture of *Physcia stellaris* (Wilken et al., 2020). The pipeline used canu v1.8 (Koren et al., 2017) for a long‐read assembly with a genome size estimation of 26 megabases. The raw contigs were corrected twice with racon v1.3.2 (Vaser et al., 2017) and subsequently polished twice with the Illumina short reads of *N. homalea* using Pilon v1.23 (Walker et al., 2014). Finally, we created a Bowtie2 (Langmead & Salzberg, 2012) database from the selected scaffolds for the mapping approach to filter for fungal RAD loci.

#### 2.2.1 RADseq library preparation and sequencing

RADseq libraries were prepared from the isolated DNA as described following Grewe et al. (2017). In summary, DNA isolations were pooled with sequence adapters (Rubin & Moreau, 2016), digested with the restriction enzyme ApeKI (New England Biolabs, Ipswich, MA, USA), and ligated using T4 ligase (New England Biolabs). All samples with compatible barcodes were pooled and selected for fragment sizes between 300 and 500 bp using the BluePippin DNA size selection system (Sage Science, Beverly, MA, USA). The pooled libraries were amplified using the REDTaq ReadyMix (Sigma‐Aldrich, St. Louis, MO, USA) prior to sequencing on an Illumina MiSeq using the MiSeq Reagent Kit v3 for 150 cycles (Illumina, San Diego, CA, USA) to produce single‐end sequences with a length of 150 bp.

#### 2.2.2 Assembly of RADseq datasets

The raw reads from the MiSeq sequencing were processed and assembled Stacks v2.3 (Rochette et al., 2019) as described earlier for metagenomic datasets of lichens (Alonso‐García et al., 2021). In short, we demultiplexed reads of individuals from the pool of raw sequence reads based on their barcodes with the script “process‐radtags.” The demultiplexed reads of each individual were aligned to the reference genome database using Bowtie2 (Langmead & Salzberg, 2012). The script “gstacks” with default parameters was used to identify SNPs in the reads aligned to the reference genome. The SNP data were then analyzed and filtered with the script “populations” considering each individual a single population. The final dataset did not allow heterozygosity at a locus (‐‐max‐obs‐het 0) and was filtered for a minimum minor allele frequency of 5% (‐‐min‐maf.05). Only the first SNP per locus (‐‐write‐single‐snp) was retained when present in at least 30% (‐R 0.3) of the individuals.

### 2.3 Phylogenomic reconstructions

**FIGURE 2:**
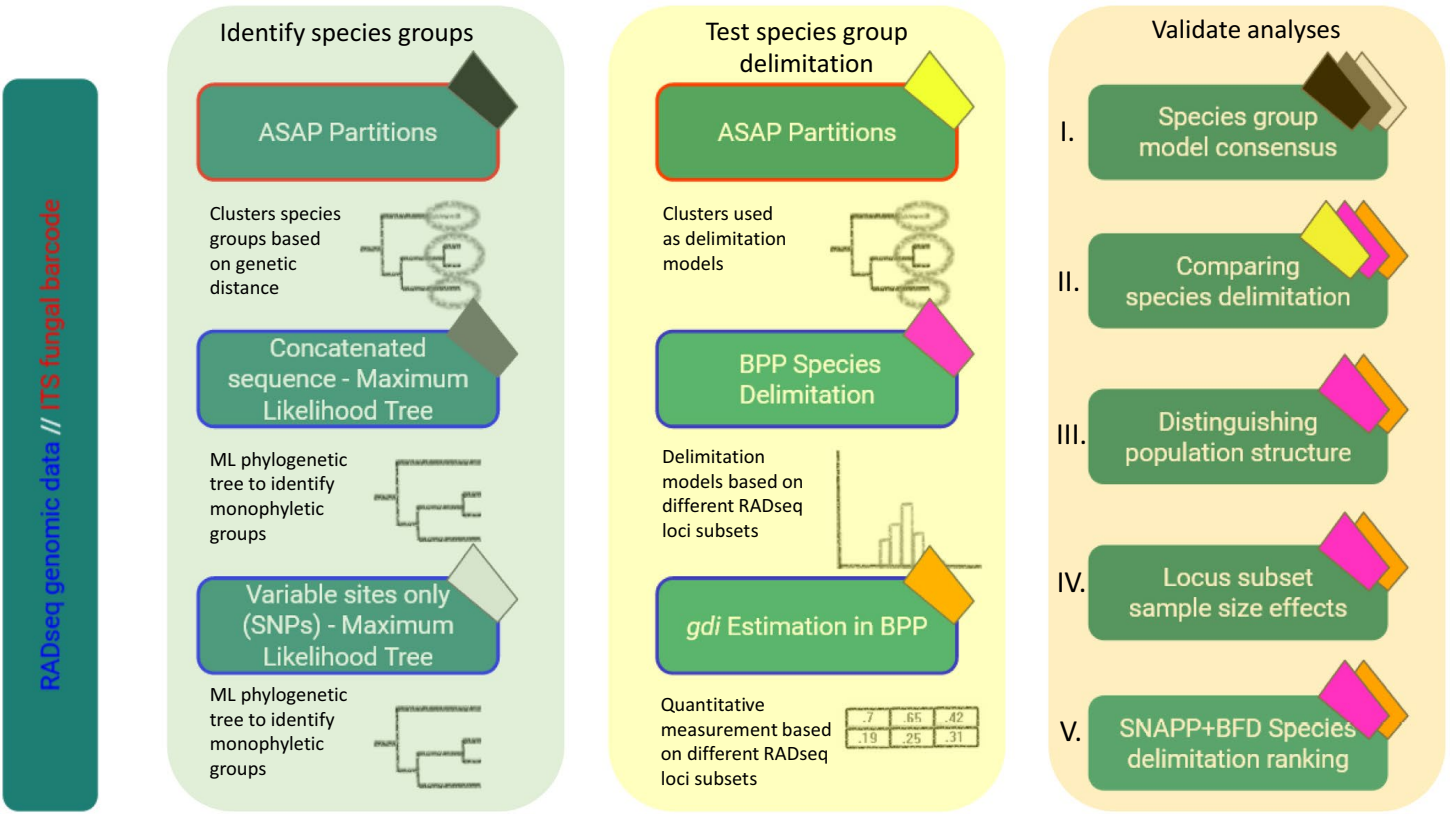
Schematic of the analyses performed in this study, separated into three categories: (1) identifying potential species groups using either genomic information (RADseq dataset, shown in blue outline) or the ITS fungal barcode (single‐locus, shown in red outline), (2) testing delimitation models in ASAP and BPP, and (3) cross‐validating analyses by comparing results from different approaches. The colored tabs represent which analyses are validated together (e.g., the three species groups models (black tab, gray tab and white tab) are compared to validate the species group model consensus). Briefly, in validation (I) we compare the species groups models from the three generated trees and/or species clusters to arrive at a consensus a priori species group model to test in the subsequent analyses. Our analyses further allow us to (II) compare the delimitation results between three different analysis types in ASAP, BPP and using the genealogical divergence index (*gdi*), (III) use the *gdi* metric to control for BPP’s bias in sometimes delimiting population structure rather than species‐level divergence, (IV) test for the effects of RADseq loci sample subsets (e.g., randomly sampled loci, or most‐informative sites, as well as the number of loci sampled) and (V) quantitatively compare the different models by attributing a Bayes factor (BF) score in SNAPP+BFD

To explore species monophyly and patterns of micro‐endemism using genome‐scale data (e.g., Wagner et al., 2013), evolutionary relationships among *Niebla* specimens were inferred from RADseq data using two strategies, (i) a supermatrix approach from the concatenated RADseq loci and (ii) a computationally efficient species tree inference approach accounting for incomplete lineage sorting (Chifman & Kubatko, 2014) using variable sites extracted from RADseq loci. Concatenation, or phylogenomic supermatrix approaches, have been shown to accurately infer relationships across a range of scenarios (Tonini et al., 2015). We inferred relationships from both the concatenated RADseq loci, and a separate dataset comprised of only variable sites (SNP dataset) as shown in Figure 2. Phylogenetic trees were reconstructed using maximum likelihood as implemented in IQ‐TREE v 1.6.12 (Nguyen et al., 2015), with 1000 ultra‐fast bootstrap replicates (Hoang et al., 2018) to assess nodal support. For the SNP dataset comprised of 198K SNPs (see results), we used the GTR+ASC to avoid overestimated branch lengths due to the SNP alignment comprised only of variable sites. For the concatenated RADseq loci dataset, comprised of 25,086 loci, total alignment length 3.6 Mb (see results), we used the GTR+I+G substitution model. Because standard concatenation approaches may return incorrect trees with high support in the presence of incomplete lineage sorting (Edwards, 2009; Kubatko & Degnan, 2007), we used SVDQuartets, as implemented in PAUP*, a method that infers relationships among quartets of taxa under the multispecies coalescent model (Chifman & Kubatko, 2014). SVDQuartets+PAUP* is computationally efficient with large genome‐scale datasets and able to accurately infer relationships under a range of scenarios (Chou et al., 2015). All possible quartets were evaluated under the multispecies coalescent tree model, and nodal support was performed using 100 bootstrap replicates.

### 2.4 Species delimitation analyses

#### 2.4.1 Single‐locus species delimitation using the standard fungal DNA barcode

To compare species boundaries inferred using the standard fungal barcoding marker with inferences from genome‐scale data, we used Assemble Species by Automatic Partitioning (ASAP; Puillandre et al., 2021). ASAP is a recently developed method that circumscribes species partitions using an implementation of a hierarchal clustering algorithm based on pairwise genetic distances from single‐locus sequence alignments (Puillandre et al., 2021). The pairwise genetic distances are used to build a list of partitions ranked by a score, which is computed using the probabilities of groups to define panmictic species. ASAP provides an objective approach to circumscribe relevant species hypotheses as a first step in the process of species delimitation. Therefore, we used ASAP to circumscribe candidate *Niebla* species from a multiple sequence alignment of the ITS barcoding region. ITS sequences generated for this study were combined with those from Spjut et al. (2020) and aligned using the program MAFFT v7 (Katoh et al., 2005; Katoh & Toh, 2008). We implemented the G‐INS‐i alignment algorithm and “1PAM/K = 2” scoring matrix, with an offset value of 0.1, the “unalignlevel” = 0.2, and the remaining parameters were set to default values. The multiple sequence alignment was analyzed using the ASAP Web Server (https://bioinfo.mnhn.fr/abi/public/asap/), with the “asap‐score” considered to select the optimal number of species partitions (Puillandre et al., 2021).

#### 2.4.2 Genomic species delimitating under the multispecies coalescent model using BPP/GDI

Incorporating genome‐scale data with the multispecies coalescent model has provided unprecedented insight into species boundaries (Flouri et al., 2018). The Bayesian program BPP includes a full‐likelihood implementation of the multispecies coalescent model (Yang, 2015), providing powerful approaches for delimiting species and populations (Degnan & Rosenberg, 2009; Knowles & Carstens, 2007; Yang & Rannala, 2010). Here, we use BPP to generate species delimitation models and cross‐validate these models in a potentially phenotypically polymorphic species in *Niebla*, see Figure 2. Due to computational limitations, we were unable to analyze the complete RADseq dataset—25,086 RADseq loci—using BPP. Therefore, we chose to explore species delimitation results using various subsets of the RADseq data. We identified the number of informative sites per RADseq locus by running the complete, partitioned dataset in IQ‐TREE v. 2.1.1 (Minh et al., 2020). Based on these results, we selected data subsets comprised of the 10 most variable loci, all loci with at least 20 informative sites (163 of the 25,086 original loci), and the 500 most variable loci. After excluding loci with no variation, we also generated data subsets comprised of 100, 500, 1000, and 5000 randomly sampled loci, in replicates of three. Finally, we included a subset of 5649 loci that included between 10 and 20 variable sites across the entire locus. To prepare these data for implementation in BPP, we used a “.fasta” file of the RAD‐Seq data, a “.txt” file with the names of the partitions of interest, and the “.txt” partition file itself. We created a custom python script (Dryad file S1), which uses those three files to output a.phy file of the RAD‐Seq data partitions of interest to make them compatible with BPP. Further, it creates a.txt file with the names of candidate species from the “.phy.” From this, a designation was made for the candidate species, for example, A1, A2, B, and C, to create the imap file for BPP. Afterward, these files were used to run “A00” and “A10” analyses in BPP (described below).

BPP analysis “A00” estimates tau (*τ*) and theta (*θ*) parameters under the multispecies coalescent model when the species phylogeny is given (Yang, 2015). Priors for the *Niebla* species population metrics, *θ* and *τ*, were estimated by running an initial naïve analysis on the 163 most variable RADseq loci in our dataset, using the *θ* and *τ* estimates of the software‐provided dataset of Asiatic brown frogs where *θ* = 0.004 and *τ* = 0.002 following Flouri et al. (2018). For each subsequent BPP analysis, the model for each data subset was specified to use *θ* and *τ* priors (*θ* = 0.0001 and *τ* = 0.00003) estimated from this initial naïve analysis, as well as using BPP’s built‐in function to re‐estimate theta during the simulation to avoid any existing taxonomic bias. Note, these priors differ widely from the parameters of the BPP analyses of Ramalinaceae reported in Spjut et al. (2020), although specific effects of different *θ* and *τ* priors on species delimitation models were not reported therein. Each analysis of the 10‐, 163‐, and 500‐most variable loci datasets was replicated six times total to account for variation in the GDI estimates, while we independently took a random sample three times and analyzed those in four replications, for a total sample of 12 per randomly sampled subset. Subsequently, we performed the “A10” analysis in BPP for species delimitation using a fixed guide tree, which provides posterior probabilities for different estimated species delimitation models using a Bayesian modeling approach (Yang & Rannala, 2010). All subsets were run for a total of 500,000 MCMC simulations, with the exception of the 5000 and 5649 subsets which were run for 100,000 MCMC simulations to avoid computational constraints. MCMC simulations were guided by a “burn‐in” of an extra 10% of the total run length, for example, 50,000 iterations for most runs. RADseq data can recover populations and recently diverged species as reciprocally monophyletic (Hou et al., 2015; Wagner et al., 2013). Recent studies have also shown that multispecies coalescent approaches to species delimitation can overestimate the number of species by delimiting population structure, rather than species‐level divergence when using genome‐scale data (Leaché et al., 2018; Sukumaran & Knowles, 2017). Therefore, 17 candidate species were initially circumscribed as reciprocally monophyletic clades consistent between the IQtree and SVDQuartets+PAUP* inferences and nested within the candidate species inferred from the single‐locus ASAP partitions, for example, phylogenetic substructure within the ASAP partitions—see results. The IQtree inference was used as the initial guide tree, collapsing nodes comprising multiple specimens within each candidate species. To assess the effect of increased coverage of RADseq loci in BPP species delimitation analyses, we used the different subsets of loci as defined above for our “A10” BPP analyses. Parameters were consistent with the “frog.ctl” found in BPP v. 4.3.0 tutorial (Flouri et al., 2018), with the exception of *θ* and *τ* priors, as mentioned above. The same 17 species phylogenetic tree prior was included in all analyses. For each dataset, MCMC chains were run for 100,000 generations, including 20,000 burn‐in iterations (Dryad file S2), and the same sample size as used in the “A00” analysis was used to control for variation between runs (six independent runs for the most informative loci datasets, and three independent subsets repeated four times each for the randomly selected loci subsets). The posterior probability of each species’ delimitation model was then pooled for an aggregate possibility between the six replicates of highly informative loci datasets, or 12 replicates of the randomly sampled loci datasets. Resulting log files were analyzed in Tracer v.1.7.1 (Rambaut & Drummond, 2005) to assess ESS values and convergence of runs.

Because genome‐scale data sets may delimit population boundaries and not species limits, we coupled our BPP analyses with the recently proposed heuristic empirical genealogical divergence index (*gdi*; Jackson et al., 2017; Leaché et al., 2018). With the proposed pattern of micro‐endemism and with putatively sympatric species common in our sampled candidate species, we used BPP’s estimates of *τ* and *θ* to calculate the *gdi* for pairwise species comparisons (Poelstra et al., 2020). We used the equation in Leaché et al. (2018), *gdi* = 1 − e^−2^ *^τ^* ^/^ *^θ^*, to calculate the probability that two sequences coalesce before reaching species divergence (*τ*) when the genealogy is traced backward in time. We used a custom python script to extract *τ* and *θ* values from the BPP analyses (Dryad file S3), treating well‐supported phylogenetic substructure in the ML topology as candidate species—see Results. The script takes in an “A00” BPP output file, calculates the *gdi* for each candidate species comparison, then writes a matrix to a.csv file. The “A00” analyses were executed for the same subsets of RADseq loci in six or 12 replicates, and *gdi* scores were calculated individually for each run and averaged across replicates. Resulting *gdi* scores for each comparison between either single species’ group or composite species’ groups were displayed in a matrix. To assess potential biases in *gdi* scores resulting from differences in the number and types of sampled RADseq loci, we visualized the distribution of *gdi* score results from the different sample subsets using a normalization function in Excel. As a rule of thumb, *gdi* values <0.2 suggest a single species and *gdi* values >0.7 suggest two distinct species, while *gdi* values within the range indicate ambiguous delimitation (Jackson et al., 2017). The resulting BPP inferred *gdi* delimitations were compared to the initial “A10” analysis performed as described above (see Figure 2 for methodological framework).

We also used an iterative, “tip‐down” approach using BPP+*gdi* scores following Jackson et al. (2017) and Leaché et al. (2018). After collapsing shallow clades comprised of morphologically identical species collected from the same location (*N. cornea* [sl16863BF & sl16857BF]; *N. dilatata* [sl16940 & sl16946]; *N*. aff. *isidiosa* [sl16775 & sl16853BF]; *N. juncosa* var. *juncosa* [sl16705 & sl16707]; and *N. juncosa* var. *spinulifera* [sl16706 & sl16719]—see Results), the topology inferred using IQtree was used as the initial guide tree, treating each terminal node as a candidate species—35 candidate species total; and specimens representing phenotype‐based species were split into multiple candidate species when not recovered as monophyletic. In BPP, the “A00” analysis was performed, and we extracted the *θ*, *τ*, and taxa labels as described above. Candidate species were collapsed into a single species group if the average of the pairwise comparisons of *gdi* scores between sister groups was ≤0.2. The process was performed iteratively until the *gdi* scores no longer suggested the joining of any candidate species in the topology. A total of ten analyses per guide tree were completed to ensure consistent results by the comparing means and standard deviations of each *gdi* score. The analyses were run for 2,000,000 iterations, with ten independent runs, calculating the *gdi* score from the mean *θ* and *τ* values.

#### 2.4.3 Bayes factor species delimitation implemented in SNAPP

As a validation of the species delimitation models derived from ASAP partitions and BPP+*gdi* (Figure 2), we used Bayes factor species delimitation (BFD*) analyses in SNAPP v.1.3 (Bryant et al., 2012). For the SNAPP analyses, 6000 variable sites evenly distributed across the dataset were extracted using a custom script and uploaded to a SNAPP template in BEAUTi v.2.6.3 (Bouckaert et al., 2019). Alternative species models were subsequently assigned based on prior species delimitation inferences from ASAP, BPP, and well‐supported clades inferred from in the ML analyses (see results) recovered within distinct ASAP partitions. Due to computational limitations in SNAPP, we divided the ML tree into three major clades, with each clade run as a separate analysis in BEAST v2.6.3 (see Dryad file S4).

Substitution rates were calculated directly in BEAUTi using the entire 6000 SNP dataset. Coalescence rate was set at 10.0 and specified to sample. Priors for both lambda and theta were set using a gamma distribution. Lambda was set to have a distribution of G(2, 500). Theta was set to have a distribution of G(1, 250). The chain length was set to 1,000,000, sampling every 1000 generations. We utilized the BFD* methods outlined by Leaché et al. (2014). Marginal likelihood values were obtained using a path‐sampling analysis in BEAST with 48 steps, chain length of 1,000,000 generations and 10% burn‐in. Resulting log files were analyzed in Tracer v.1.7.1 (Rambaut & Drummond, 2005) to assess ESS values and convergence of runs. Each model was run four times and the marginal likelihood value was averaged across each clade in order to further ensure convergence.

#### 2.4.4 Modeling the potential effects of migration and genetic drift

The program TreeMix v 1.13 (Pickrell & Pritchard, 2012) was used to model the potential effects of migration and genetic drift. Utilizing the “populations” script again, the data input into “gstacks” was transformed into a format suitable for this program (‐‐.treemix). All exemplars from the original maximum likelihood tree (Dryad file S5) were included in the “.treemix” file, and sl16502 was used as the specified outgroup in the analysis as the program is limited to one. Each outgroup was tested, but this made no real difference in evidence for migration or genetic drift. Topologies were visualized using R (R Core Team, 2019) via the plotting_funcs.R script from the TreeMix package and edited in Adobe Illustrator v24.3.

## 3 RESULTS

The number of Illumina reads for each sample varied from 58,930 to 1,151,143, with an average of 326,580 sequences per sample, with 7.3% to 75.3% of reads mapped to the *Niebla* reference using Bowtie2 (Dryad file T1). The number of *Niebla* RADseq loci (within‐sample clusters) that STACKs generated from these sequences ranged from 2534 to 39,941 (mean = 22,373 loci/sample). Our final RADseq dataset resulted in a total of 25,086 143‐bp loci. The total length of the concatenated RADseq loci was 3.6 Mb, including 198K variable sites.

### 3.1 Phylogenetic reconstructions

**FIGURE 3:**
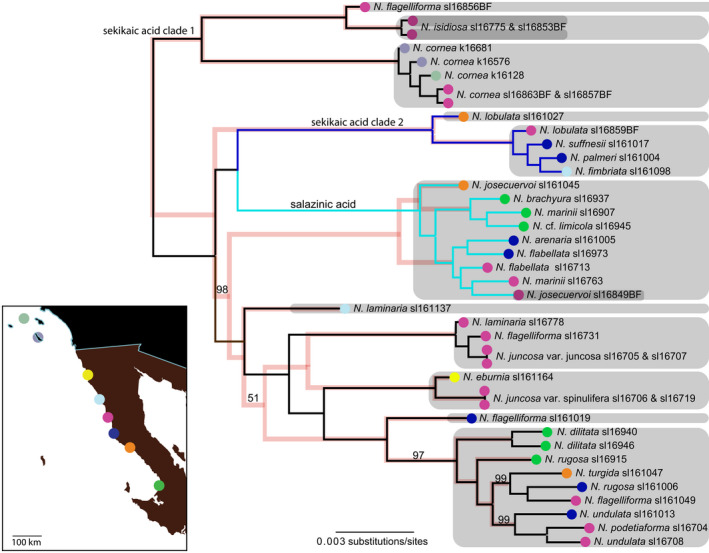
ML topology inferred from 298,000 variable sites distributed across over 25,000 RADseq loci. Pink “shadow” tree is the species tree inferred under the multispecies coalescent model in PAUP+SVDquartets. Colors at tips correspond to sampling sites in map in bottom‐left panel. Chemical clades are indicated with colored branches on the ML topology: Black branches indicate lichens producing divaricatic acid (four clades), dark‐blue branches indicate lichens producing sekikaic acid (two clades); and light blue branches indicate lichens producing salazinic acid. Gray boxes correspond to ASAP partitions from analyses of the standard DNA‐barcoding marker for fungi (ITS), with double shaded tips representing separate ASAP partitions that were combined for all subsequent analyses. Outgroup—*Vermalicinia* specimens—not shown. Values shown at nodes indicate bootstraps support, no value printed indicates a bootstrap support value of 100

The ML topologies inferred from both the concatenated RAD‐Seq data and the SNP data recovered identical, well‐supported topologies (Figure 3). Major clades recovered here coincided with the production of distinct secondary metabolites, although specimens producing depsides of either sekikaic acid or divaricatic acid were each recovered in two polyphyletic clades (Figure 3) with a much larger divaricatic acid clade sister to the depsidone (nonterpenoid) clade, or a clade divided into sekikaic acid and depsidone subclades. Phenotypically circumscribed species represented by multiple samples were not recovered as monophyletic except those identified as *N. cornea*; they were collected from the Channel Islands, California, USA, and from near San Quintín, Baja California, Mexico (Dryad file T1; Figure 3). Additionally, specimens collected in close proximity having the same chemotype—but differentiated morphologically as distinct species or varieties—occurred in different clades (Figure 3); examples are divaricatic acid specimens that include saxicolous sl‐16775 (*N. *aff. *isidiosa*) and sl‐16778 (*N. laminaria*), sl‐16705 (*N. juncosa* var. *juncosa*) and sl‐16706 (*N. juncosa* var. *spinulifera*), sl‐16707 (*N. juncosa* var. *juncosa*), and sl‐16708 (*N. undulata*). Those specimens evidently growing in close proximity differentiated as species more by their secondary metabolites—obviously in different clades—include, for example, terricolous sl‐161004 (*N. palmeri*, sekikaic acid), sl‐161005 (*N. arenaria*, salazinic acid), and saxicolous sl‐161006 (*N. rugosa*, divaricatic acid). As many as eight species have been recognized at a location (Spjut et al., 2020). SVDQuartets+PAUP* resulted in a generally well‐supported topology, although in some cases backbone relationships differ strikingly from the ML reconstruction (Figure 3; Dryad file S5). In other cases, morphologically circumscribed species occurring in the same locality may comprise multiple candidate species‐level lineages, for example, specimens identified as *N. flabellata* and *N. lobulata*. Finally, the TreeMix analysis showed no significant evidence of migration or genetic drift in the study organisms (Dryad file S8).

### 3.2 Species delimitation

#### 3.2.1 ASAP species delimitation using the standard fungal barcode

A maximum of 32 species partitions were inferred from the combined ITS dataset (ITS sequences generated for this study+ITS sequences reported in Spjut et al., 2020) using ASAP, 13 of which were represented by the samples selected for RADseq. The majority of ASAP partitions containing at least two samples were comprised of morphologically/chemically polymorphic specimens e.g., specimens identified as different species (Figure 3). The ASAP partitions inferred from the standard fungal barcode marker largely coincided with reciprocally monophyletic clades inferred for RADseq data in the SVDQuartets+PAUP* and ML topologies (Figure 3). Specimen sl16849BF (*Niebla* “sp. nov.”) was inferred as a distinct species partition in ASAP but was combined with another partition comprising the remainder of the specimens in the same clade for all subsequent analyses. This specimen was morphologically similar to *N. versiforma* but contains salazinic acid, rather than divaricatic acid (with triterpenes), and represents a putative undescribed species. Specimens sl16775 & sl16853BF, both representing isidiate *Niebla* specimens (Figure 1e,k) and collected from the same locality, were inferred as separate ASAP partitions but were combined for all subsequent analyses tentatively identified as *N*. *isidiosa* (Figure 3).

#### 3.2.2 Species delimitation using RADseq loci under the multispecies coalescent using BPP

**FIGURE 4:**
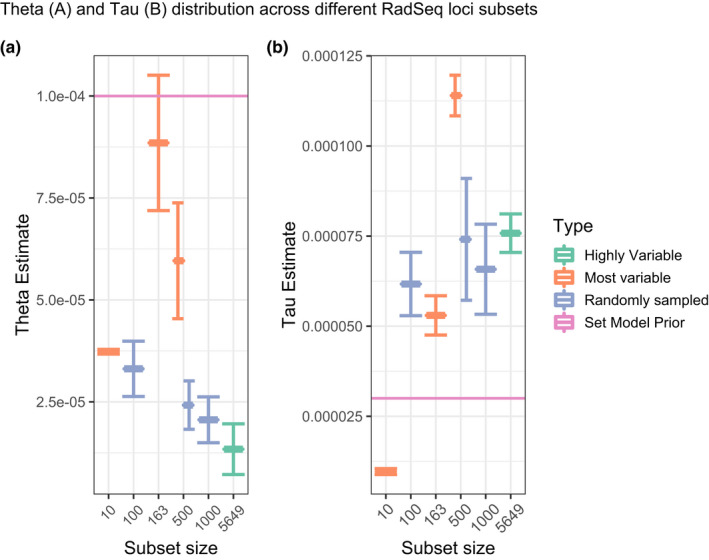
Theta and Tau posterior estimates across different RadSeq loci subsets. (a) Mean Theta and standard deviation are estimated across all 17 *a priori* species nodes from each simulation. (b) Mean Tau and standard deviation are estimated from only the ancestral node across the different simulations. The pink lines indicate the *a priori* estimate of Theta and Tau used for all simulations

**FIGURE 5:**
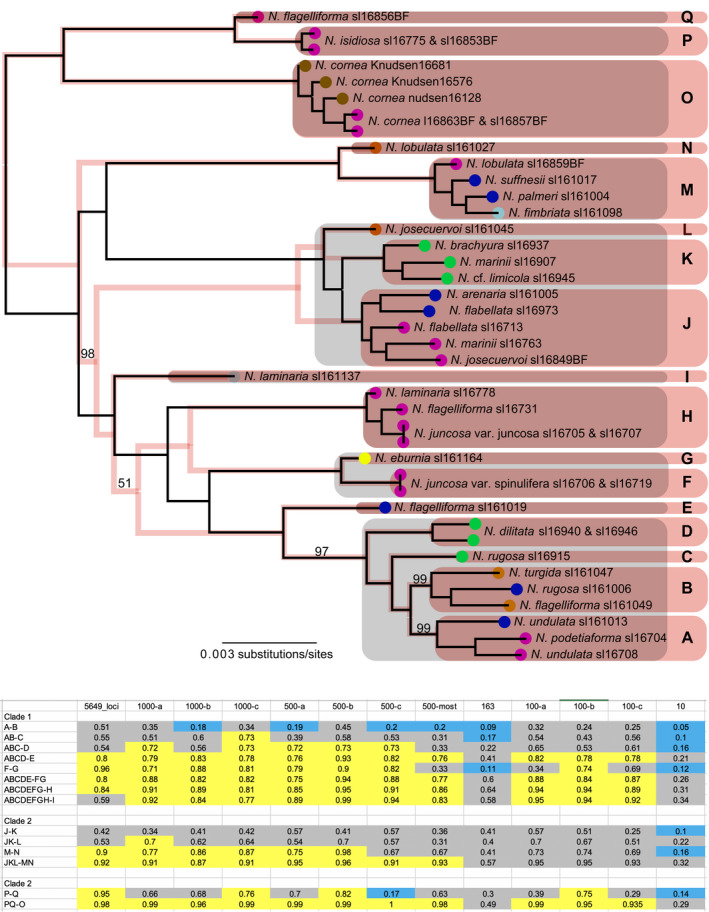
Candidate species and species delimitation analyses using BPP+*gdi*. (a) Clades labeled “A” through “Q” and highlighted in pink boxes, represent candidate species defined based on phylogenetic subdivision inferred from the ML and SVDquartets+PAUP tree inferences (Pink “shadow” tree is the species tree inferred under the multispecies coalescent model in SVDquartets+PAUP); tips collapsed in the “tip‐down” BPP+*gdi* approach are highlighted with purple boxes. (b) Heuristic *gdi* values inferred from different candidate species comparisons—see candidate species in panel “a”—and using different subsampled RADseq datasets (5649 variable RADseq loci; three random subsets of 1000 variable loci; three random subsets of 500 variable loci; the 500 most variable loci; the 163 most variable loci; three random subsets of 100 variable loci; and the ten most variable loci); following Jackson et al. (2017), *gdi* scores ≤0.2 indicate a single species (highlighted in blue), *gdi* scores ≥0.7 indicate distinct species highlighted in yellow, and status for comparisons with *gdi* scores between 0.2 and 0.7 are ambiguous (highlighted in gray)

**FIGURE 6:**
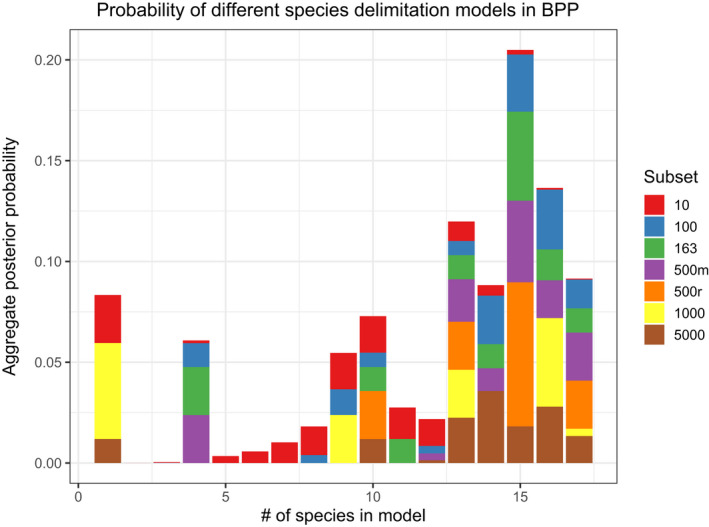
Probability of different species delimitation models—species “A”—“Q,” Figure 3—inferred using the “A10” species delimitation model in the program BPP. The results are summarized from all analyses of RADseq data subsets, with the *Y*‐axis shows the probability, in aggregate, for the inferred number of species

Estimates of *θ* values under the multispecies coalescent model—analysis “A00” in BPP—were on the same order of magnitude regardless of different RADseq loci subsamples, although standard deviation was much greater in the 163‐ and 500‐most variable loci data subsets (Figure 4, exact values reported in Dryad file T2) and reported posterior theta estimates were generally lower than the model prior. Greater variation in *τ* values was observed depending on the subsampled RADseq loci (Figure 4) but all posterior estimates fell well within an order of magnitude difference from the model prior. Results using the same subset of RADseq loci generally converged on similar *gdi* estimations. Species delimitation using a fixed guide tree—analysis “A10” in BPP—and the candidate species shown in Figure 5 inferred up to 17 species, the maximum subdivision specified in the guide tree. Aggregate probabilities were calculated across all runs by averaging the posterior probability of each species model and resulted in the highest aggregate probabilities across the 13–17 species range, with a 15 ‐species model being most likely across all analyses (Figure 6). However, the results of the “A10” species delimitation analyses in BPP varied across both subsets of data and independent runs, with the randomly sampled subsets showing less proclivity of an unrealistic one‐species model (Dryad file T2; Dryad file S6).

While the candidate species collapsed under each analysis varied widely, the most frequent candidate species groups that were collapsed included: “O,” “P,” and “Q” collapsed into 1 or 2 species; “J,” “K,” “L,” “M,” and “N” were frequently collapsed into 1, 2, or 3 different species groups; and “A,” “B,” “C,” and “D” which were frequently collapsed into 1 or 2 species groups, sometimes including “E” (Figure 5).

#### 3.2.3 Species delimitation validation using *gdi* scores

The *gdi* scores inferred from parameters under a multispecies coalescent model—analysis “A00” in BPP—generally did not provide justification for collapsing any nodes in the 17‐species model—based on 17 well‐supported clades identified in the ML topology, the maximum subdivision specified in the guide tree (Figure 5; Dryad file S6), suggesting that our sampling includes at least 17 species‐level lineages. However, in the analyses of the 10 and 163 most informative RADseq loci, a number of candidate species were collapsed based on *gdi*. In the 10‐loci subset, candidate species “A,” “B,” “C,” and “D” were combined (*gdi* scores <0.2), as were candidate species “F” and “G,” candidate species “J” and “K,” candidate species “M” and “N,” and finally candidate species “P” and “Q” (Figure 5), for a resulting model with 10 delimited species. In the 163‐loci subset analysis, candidate species “A,” “B,” and “C” were collapsed together, as well as “F” and “G” into a 14‐species model. The *gdi* scores calculated from all other analyses suggested maintaining the 17‐species model. In the “tip‐down,” iterative approach, *gdi* scores supported collapsing a number of tips resulting in a 29‐species model from the original 35 tips after combining morphologically similar specimens from the same location (Figure 5; Dryad file S7). Collapsed specimens included two of the three specimens identified as *N. cornea* from the Channel Islands with two specimens from Baja California (in clade “O”), specimens identified as *N. fimbriata*, *N. palmeri*, and *N. suffnessii* (in clade “M”), *N. flagelliforma* with *N. juncosa* var. *juncosa* (in clade “H”), and *N. flagelliforma* with *N. rugosa* (in clade “B”) (Figure 5).

**FIGURE 7:**
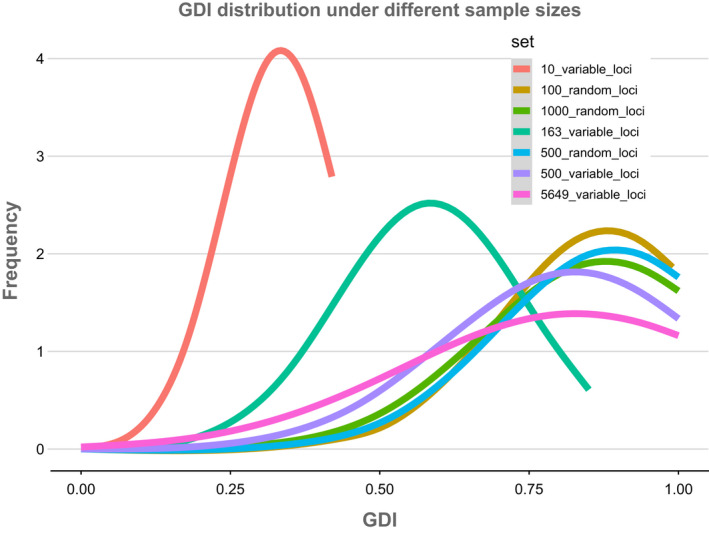
Distribution of *gdi* scores estimated from different subsets of RADseq loci (143 bp each)

To further characterize the impact of data subsampling on *gdi* scores, we report average *gdi* scores across all nodes for each of the RADseq data subsets. The 10‐ and 163‐locus datasets comprising the most variable sites had lower *gdi* scores when compared with the remaining subsets (Figure 7). Average *gdi* values generally increased with an increased coverage of genetic markers, although using the most informative markers rather than a random subset seems to limit this trend. This trend is primarily caused because the *τ* estimate is much higher when estimated using only the most variable loci, with the exception of a very small dataset of 10 loci. Estimates for *θ* of each individual candidate species were also higher when using the most informative subsets, but so is the standard deviation of the estimated and *θ* values.

#### 3.2.4 BFD* implemented in SNAPP

In all cases, BFD* delimited more putative species than the single‐locus ASAP analysis (Figure 3; Dryad file S4). BFD* analyses supported the most divisive species delimitation models in two of the three subclades—clades “1” and “3,” and the intermediate species delimitation model for “Clade 2.” The intermediate species delimitation model for “Clade 2” directly matches the a priori species group delimitation used for the BPP and *gdi* analyses, as do the second‐best models for “Clade 1” and “Clade 2”. The “Clade 1” model tested to validate the results from the *gdi* and A10 analyses where species groups A, B, C, and D were collapsed had a much lower reported likelihood, as did the analyses where P and Q (Clade 3) and J, K, and L (Clade 2) were collapsed (see Figure S4). Marginal likelihood values across each of the four independent runs were largely consistent but tended to be slightly more variable with an increasing number of species.

## 4 DISCUSSION

Our study provides the first genome‐scale perspective into diversification of the lichen‐forming fungal genus *Niebla*—endemic to the Pacific coast of Baja California, highlighting dynamic speciation in this clade of symbiotic fungi. Our data support the perspective of a species‐rich genus and that many species are indeed sympatric, occurring in close association. However, single species populations have also been recognized, mostly near the range limits of the genus (Spjut, 1996; Spjut et al., 2020). Although candidate species inferred from our genome‐scale data outnumbered current phenotype‐based species circumscriptions, this is to be expected if *Niebla* populations in Baja California fits a model of microendemism (Spjut et al., 2020). Furthermore, the lack of evidence for significant migration or genetic drift from the TreeMix analysis support said model. In fact, migration was essentially nonexistent and the drift parameter was orders of magnitude less than those reported in other study systems (Brandrud et al., 2017; Pickrell et al., 2012).

On the other hand, morphological parameters applied to distinguish species could be revised to agree with molecular data assuming there are no cryptic species. However, cryptic species are evident for *N. homalea* at the putative type locality (Spjut et al., 2020; 17801 & 17802), *N. dilatata* (sl‐16946 & 16947—not cited in this study), and other phenotypic species. Chemical variation in lichen metabolites also need further study, such as triterpenes in *N. lobulata* (Spjut, 1996) that may correlate with species differences (Spjut et al., 2020), and isodivaricatic acid found in type specimens of *N. eburnea* (Spjut‐Rakatondraibe comm., Oct 2016) instead of divaricatic acid as reported for *N. homalea* from the putative type locality (Zhang et al., 2020). Nevertheless, similar to an earlier study using multilocus sequence data (Spjut et al., 2020), we found that robust, consistent species delimitations were difficult to infer, even with thousands of RADseq loci. Below we discuss the implications of our findings in relationship to the challenge of species delimitation and characterizing diversity in *Niebla*.

### 4.1 Species delimitation in the “messy middle”

Differences in species delimitation studies, ranging from the number and type of loci selected, how candidate species are initially identified, for example, morphology‐based vs. sequenced‐based, to empirical analytical approaches, can result in discrepancies in the inferred species boundaries (Carstens et al., 2013; Luo et al., 2018). Furthermore, differences in diversification histories likely require different types of data and different analytical approaches in order to accurately delimit species boundaries (Camargo & Sites, 2013; Leavitt et al., 2015). With an increasing number of species inferred from phylogenomic datasets, the problem of inferring population structure versus actual species boundaries becomes acute (Leaché et al., 2018). Therefore, how researchers select the appropriate number of loci and analytical approaches becomes increasingly important as analyses continue to increase in scale. Recognizing that any species delimitation approach infers hypotheses of species boundaries, rather than providing the ultimate answer/solution, is imperative (Matute & Sepúlveda, 2019). Furthermore, species delimitation studies with sparse species‐level sampling—limited to one to several samples representing candidate species—may lead to inadequate representation of intraspecific variability, resulting in incorrect inferences of species boundaries (Lim et al., 2012). Initial surveys with relatively sparse sampling, such as some of the candidate species inferred in this study, can help direct future research, highlighting areas where dense sampling at population levels in a group of closely related species can help elucidate more robust species boundaries (Zheng et al., 2017). Furthermore, inferences from genomic data must also be considered in conjunction with other information (Sukumaran et al., 2021). Hence, integrating independent lines of evidence with densely sampled population‐level phylogenomic datasets can provide increasingly robust hypotheses of species boundaries (Fujita et al., 2012; Yang et al., 2019) and will be essential for characterizing diversity in *Niebla*.

The results of our study highlight the problem of delimiting species, particularly in groups such as *Niebla*, with complex, recent phylogeographic histories. Despite the informed perspective from genome‐scale data and long‐term investigations of phenotype‐based circumscriptions, species boundaries in *Niebla* lichens are far from being resolved. While differences in the number of delimitated candidate species in *Niebla*, depending on the analyzed loci and species delimitation analysis, resulted in no clear favored species delimitation model, our analyses supported high species‐level diversity in *Niebla*. Both BPP species delimitation analyses and BFD* analyses implemented in SNAPP provided strong evidence for multiple species‐level lineages in ASAP species partitions inferred from the standard fungal barcoding marker, the ITS region (Figures 3 and 5; Dryad file S4). The “tip‐down” approach using BPP+*gdi* scores (Jackson et al., 2017; Leaché et al., 2018), supported the highest number of candidate species from our dataset, a 29‐species model (Figure 5). The BFD* analyses also generally favored the most divisive species delimitation models (Dryad file S4), although independent runs did not always converge with the more divisive species models. While the BFD* provides a direct, quantitative method for comparing species models with increasing numbers of sampled loci and candidate species, computational limitations may constrain the utility of this methodological approach (Leaché et al., 2014). In this study, we attempted to circumvent these limitations by subdividing the topology in order to analyze computationally feasible data subsets. Despite those efforts, convergence among independent runs analyzing subclades remained an issue in models with even a modest number of species (Dryad file S4).

While the heuristic *gdi* criterion for species delimitation provides a metric to guide the distinction of population structure from species boundaries (Jackson et al., 2017; Leaché et al., 2018), we show here that locus selection can have a substantial impact on *gdi* scores. In the empirical scenario of *Niebla*, selecting limited, but highly variable, RADseq loci resulted in the lowest *gdi* scores, specifically in the 10‐ and 163‐marker datasets (Figure 7). In contrast, randomly selected RADseq loci, resulted in largely consistent, higher *gdi* scores; and these *gdi* scores were more consistent with the 500‐most variable RADseq loci dataset. These results suggest that when using short RADseq loci, it is important to maximize the number of analyzed loci if possible, but that a relatively low number of randomly sampled loci can result in consistent inferences. These observations are in line with results of other simulations showing a limited effect of selection pressures in affecting the results of multispecies coalescent models (Wascher & Kubatko, 2020). Higher numbers of loci under selection have been shown to introduce a bias toward larger *τ* values and underestimated *θ* values up to a factor of 20 (Adams et al., 2018), yet our computed posterior estimates of these variables remained remarkably consistent across most subsets (Figure 4), indicating that the number of loci under selection even in the most variable loci subsets was likely low. This is also reflected in the species delimitation results where variation is present but most simulations converge on a 13–15 species model (Figure 6). We suggest that when rates of selection cannot be easily calculated running simulations with different loci subsets is a good substitute to test the robustness of the multispecies coalescent model used.

The heuristic *gdi* criterion may also be biased in cases where a population was established by a few founder individuals and *N_A_* and *θ_A_* may be very small. Because the criterion depends on the population divergence time relative to population size (2*τ*/*θ_A_*), the use of *gdi* may lead to claims of species status even if the populations are recently diverged (Leaché et al., 2018). We speculate that this may be the case for *Niebla* populations in Baja California. Here, we inferred recent divergence among *Niebla* lineages, and it would not be unreasonable to suspect that independent populations were founded by few individuals. If this is the case, it may be necessary to consider both the absolute population divergence, as well as the population divergence relative to population size (Leaché et al., 2018; Yang & Rannala, 2010). Finally, given the large range of indecision using the *gdi*, it may not be possible to infer with confidence species boundaries given the nature of the speciation process in these lichen‐forming fungi.

### 4.2 Concordance between the standard DNA barcoding marker and clades inferred from phylogenomic data

The standard DNA barcoding marker—the ITS—has been a powerful tool for understanding species diversity in lichen‐forming fungi (Lücking et al., 2020; Schoch et al., 2012), although issues with using this marker in some clades have been demonstrated (Pino‐Bodas et al., 2013). In taxonomically challenging groups, the ITS can provide an important first pass for grouping closely related species (Leavitt et al., 2013; Lücking et al., 2014; Moncada et al., 2020). While the taxonomic implications of DNA barcoding approaches remain unsettled (see Meier et al., 2021), our results show that species partitions inferred from the ITS marker coincide with clades comprised of closely related species‐level lineages in *Niebla* inferred from phylogenomic data (Figure 3). These results suggest that in this speciose genus, the ITS can play a crucial role in assigning specimens to species complexes and provide guidance for future studies characterizing species‐level diversity. However, our analyses of ITS alignments indicate that the ITS alone is not able to diagnose species‐level lineage in *Niebla*. Extensive sampling of specimens representing ITS‐delimited species, coupled with genome‐scale data, will likely be essential to robustly characterize diversity in this genus. Similarly, focusing on specific habitats in Baja California supporting diverse *Niebla* communities, including Mesa Camacho, Mesa Santa Catarina, ridges between Punta Santa Rosalillita and Punta Negra, and Morro Santo Domingo, will be crucial to infer diversification processes in important genus of lichen‐forming fungi occurring only in coastal fog deserts.

While the results of our analysis are somewhat ambiguous in terms of species delimitation, they provide a valuable perspective of the use of these empirical species delimitation methods in a nonmodel system. Furthermore, they add to the growing body of literature on the all‐too‐often “messy” species boundaries in organisms that further complicate our understanding of their complex evolutionary history. Future work should build upon the hypotheses presented here to add additional lines of evidence for specific species models in order to resolve the taxonomy in this group. Lichen‐forming fungi are notoriously difficult to define in terms of species delimitation, and in some cases, traditional morphological and chemical characters used for taxonomy fail to reflect natural lineages (Lumbsch & Leavitt, 2011). Nevertheless, more thorough chemical investigations may clarify some species in *Niebla* and ecological and paleoclimatic features should be considered in future work on their speciation processes (Sukumaran et al., 2021). Finally, to resolve taxonomic issues in *Niebla*, species will need to be assessed from fresh material collected at the type locality.

## CONFLICT OF INTEREST

The authors declare that they have no conflict of interest.

## AUTHOR CONTRIBUTIONS

**Jesse Jorna:** Conceptualization (equal); formal analysis (equal); investigation (equal); methodology (equal); software (equal); visualization (equal); writing – original draft (equal); writing – review & editing (equal). **Jackson B. Linde:** Conceptualization (equal); formal analysis (equal); writing – original draft (equal); writing – review & editing (equal). **Peter C. Searle:** Conceptualization (equal); formal analysis (equal); methodology (equal); writing – original draft (equal); writing – review & editing (equal). **Abigail C. Jackson:** Conceptualization; formal analysis; methodology; writing – original draft (equal); writing – review & editing (equal). **Mary‐Elise Nielsen:** Conceptualization; formal analysis; methodology; writing – original draft (equal); writing – review & editing (equal). **Madeleine S. Nate:** Conceptualization; formal analysis; writing – original draft (equal); writing – review & editing (equal). **Natalie A. Saxton:** Conceptualization; formal analysis; writing – original draft (equal); writing – review & editing (equal). **Felix Grewe:** Conceptualization; data curation; formal analysis; funding acquisition; methodology; resources (equal); software (equal); validation (equal); writing – review & editing (equal). **María de los Angeles Herrera‐Campos:** Investigation; methodology; resources (equal); writing – review & editing (equal). **Richard W. Spjut:** Investigation; methodology; validation (equal); writing – original draft (equal); writing – review & editing (equal). **Huini Wu:** Methodology; writing – review & editing (equal). **Brian Ho:** Methodology. **H. Thorsten Lumbsch:** Conceptualization (equal); methodology; writing – review & editing (equal). **Steven D. Leavitt:** Conceptualization (equal); data curation (equal); formal analysis (equal); funding acquisition (equal); investigation (equal); methodology (equal); project administration (equal); resources (equal); software (equal); supervision (equal); visualization (equal); writing – review & editing (equal).

## Supporting information

Supplementary_ S1_script1

Supplementary_ S2_script2

Supplementary_ S3_script3

Supplementary_ S4_snapp_species_v1

Supplementary_ S5_topologies_IQtree_svdquartets

Supplementary Material

Supplementary Material

Supplementary_ S8_treemix

## Data Availability

All supporting files are available on Dryad: https://doi.org/10.5061/dryad.9ghx3ffh4. Dryad file T1. Information about the specimens collected in Baja California including initial taxonomic assignment and GPS location. Dryad file T2. Species delimitation results from the “A10” analysis in BPP and theta and tau parameter estimates from the “A00” analysis in BPP under different size loci subsets and resulting average gdi values. The most likely delimitation model is displayed in underscore with the range of all estimations assigned some posterior probability in brackets. The fraction is the averaged posterior probability of the most likely delimitation model across the entire data subset. Posterior probabilities were averaged across the repeated analyses (*n* = 6 for most informative, *n* = 12 for random subsets) as results were often different between individual simulations. Theta is averaged for each individual species’ group in the 17 species models across the entire data subset, and tau here is given for the most recent common ancestor for all species (Ancestral node). Dryad file S1. “Script 1”—custom python script for concatenating FASTA sequences into BPP‐style files using a “.fasta” file of the RAD‐Seq data, a “.txt” file with the names of the partitions of interest, and the “.txt” partition file itself. Those three files are used to output a.phy file of the RAD‐Seq data partitions of interest to make them compatible with BPP. Further, it creates a.txt file with the names of candidate species from the “.phy” file. Dryad file S2. “Script 2”—control file for the A10‐type analysis in BPP. Dryad file S3. “Script 3”—custom python script to extract *τ* and *θ* values from the BPP analyses to calculate *gdi* scores directly from BPP “A00”‐type analysis output. Dryad file S4. Results of the SNAPP+BFD* analyses. Page 1—the five different species delimitation models for “clade 1,” ranging from 3 to 16 species assessed; the 16‐species model was selected using Bayes factors (BF). Page 2—the three different species delimitation models for “clade 2” and “clade 3”; in “clade 2,” the 5‐species model was selected over the 2‐ and 13‐species models; in “clade 3,” the 7‐species model was selected over the 2‐ and 3‐species models. Page 3 – BF scores and rankings from the clade‐specific SNAPP+BFD* analyses. Dryad file S5. Side‐by‐side comparison of the ML topology inferred using IQtree and the species tree inferred using PAUP+SVDquartets (summarized in Figure 3). Values at nodes indicate bootstrap support when less than 100%. Dryad file S6. *gdi* scores and MCMC runs from the “A10” species delimitation analyses using different subsets of RADseq loci. Dryad file S7. Results from the BPP +gdi *“*tip‐down” species delimitation approach. Dryad file S8. Treemix topology inferred from RADseq data. Note the lack of migration and the insignificant level of genetic drift suggested by the analysis. All RADseq reads are deposited in NCBI Short Read Archive under project # pending.
