# Supplementary material for: Species boundaries in the messy middle—A genome‐scale validation of species delimitation in a recently diverged lineage of coastal fog desert lichen fungi
Source: Ecol Evol. 2021 Dec 19;11(24):18615–32. doi: 10.1002/ece3.8467 (PMC8717302; doi:10.1002/ece3.8467)
Supplement: Supplementary file 4 — Supplementary_ S4_snapp_species_v1 [file ECE3-11-18615-s008.pdf]

C1.IQ3 / ASAP 7.3   3 species

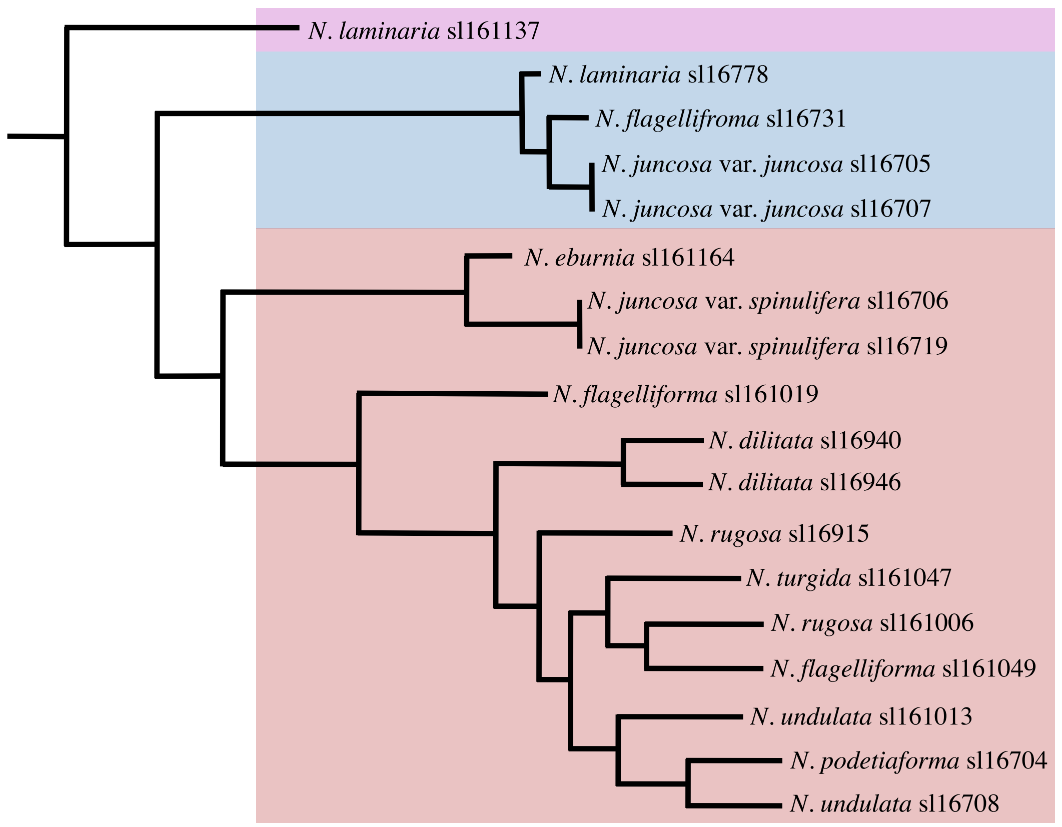

C1.IQ5 5 species

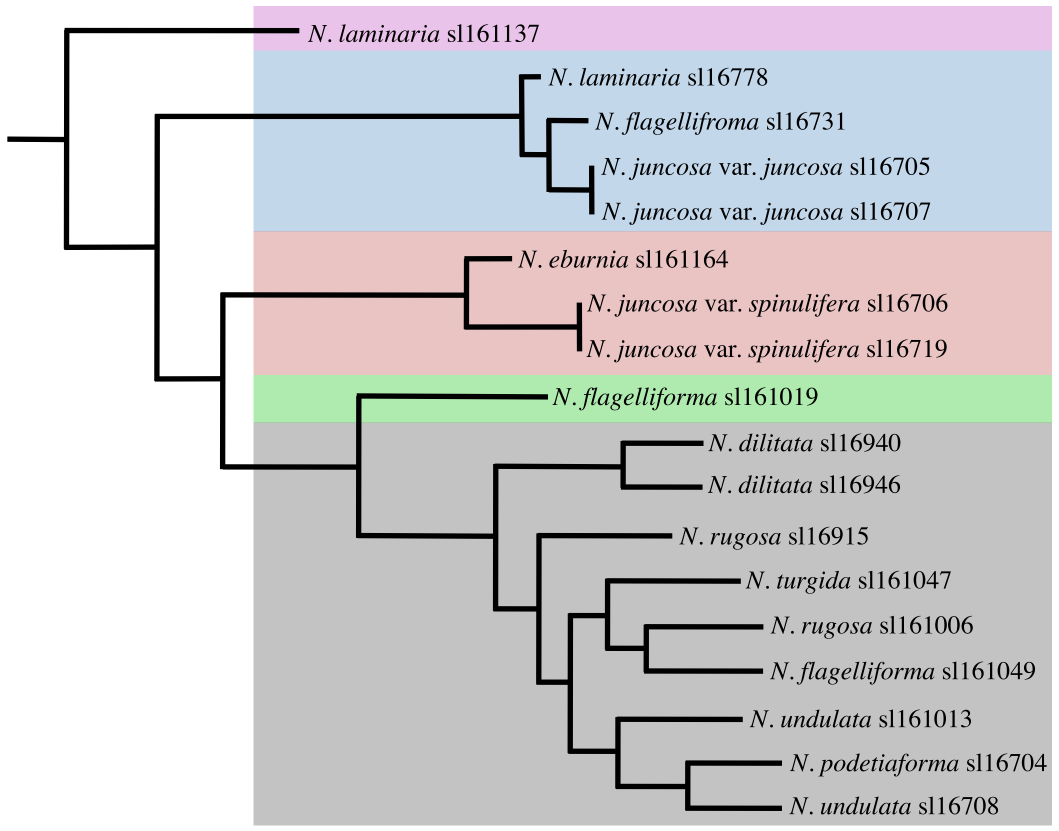

C1.IQ9 9 species

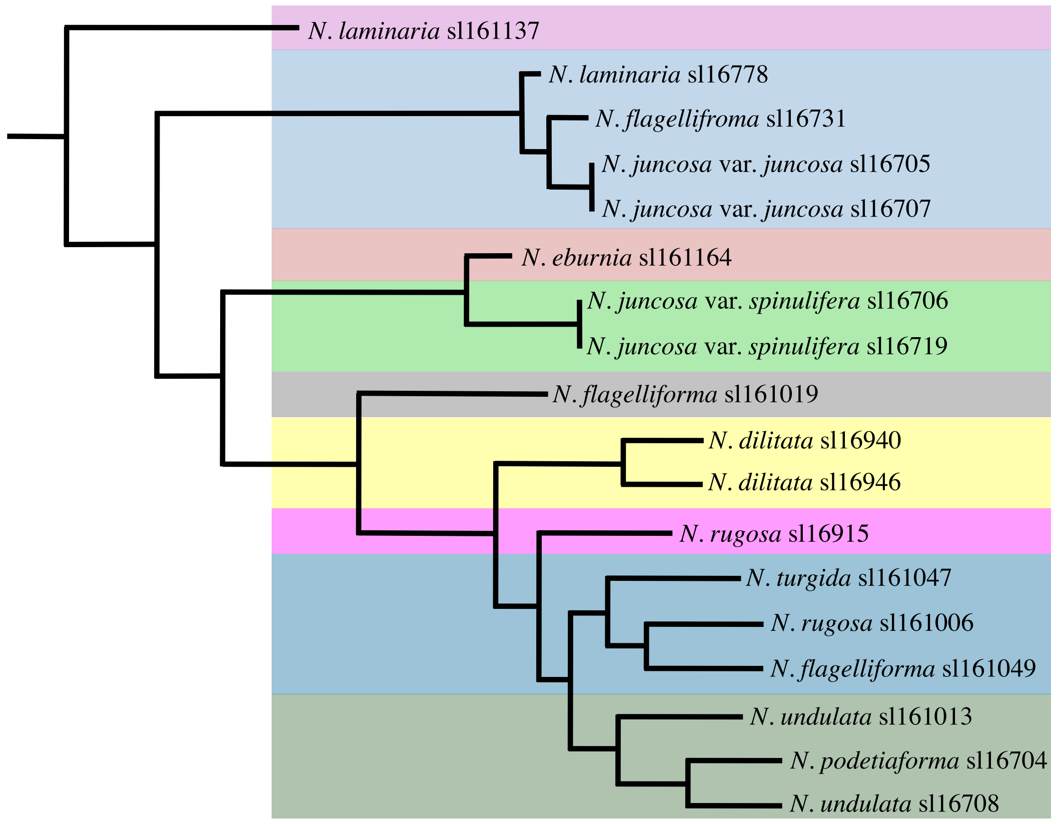

C1.ASAP9.5 5 species

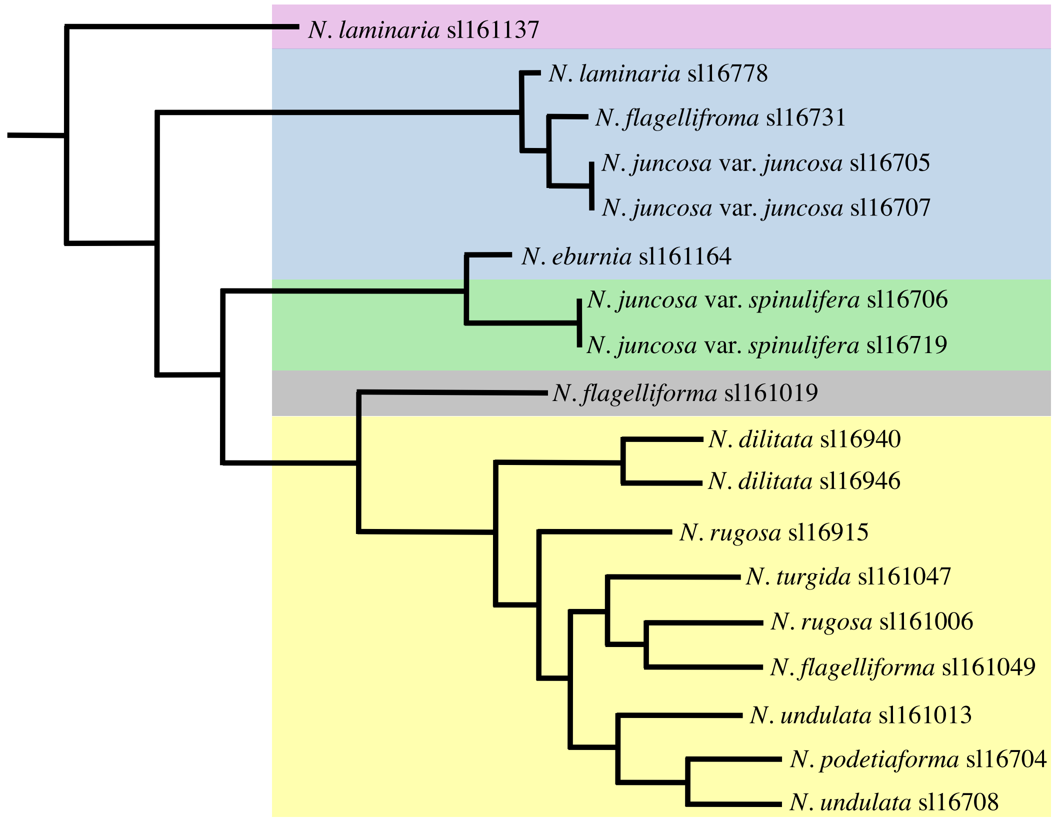

C1.BPP16 16 species

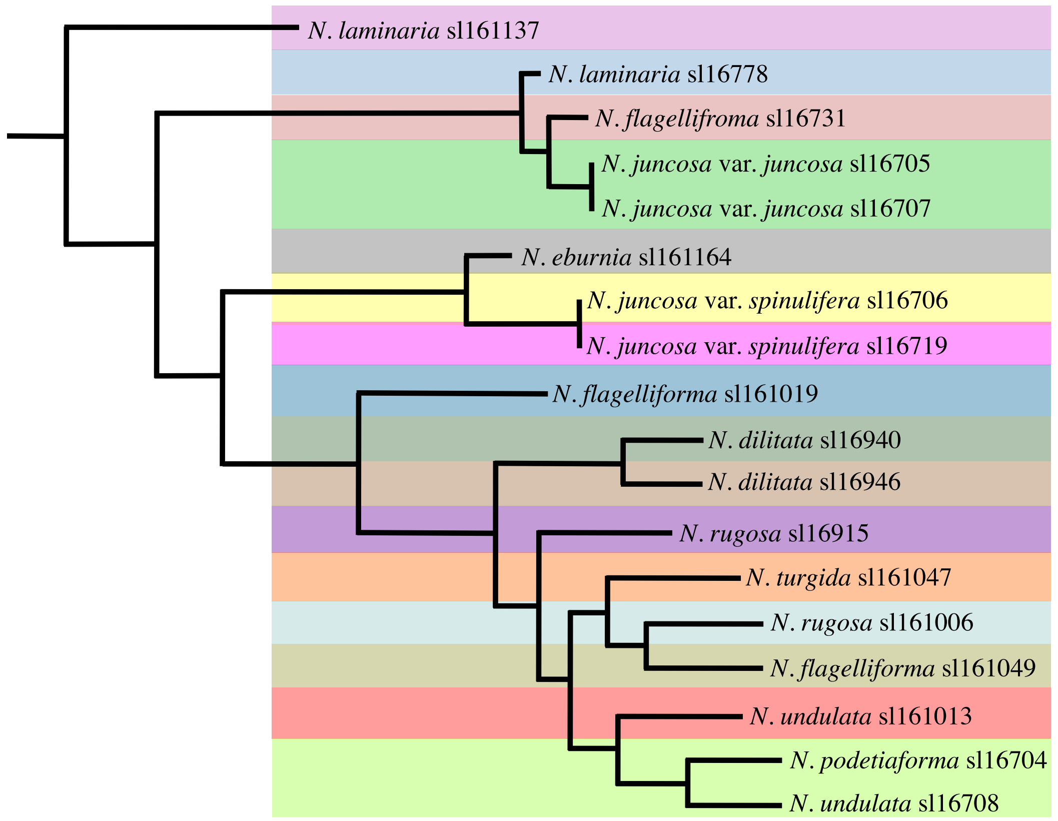

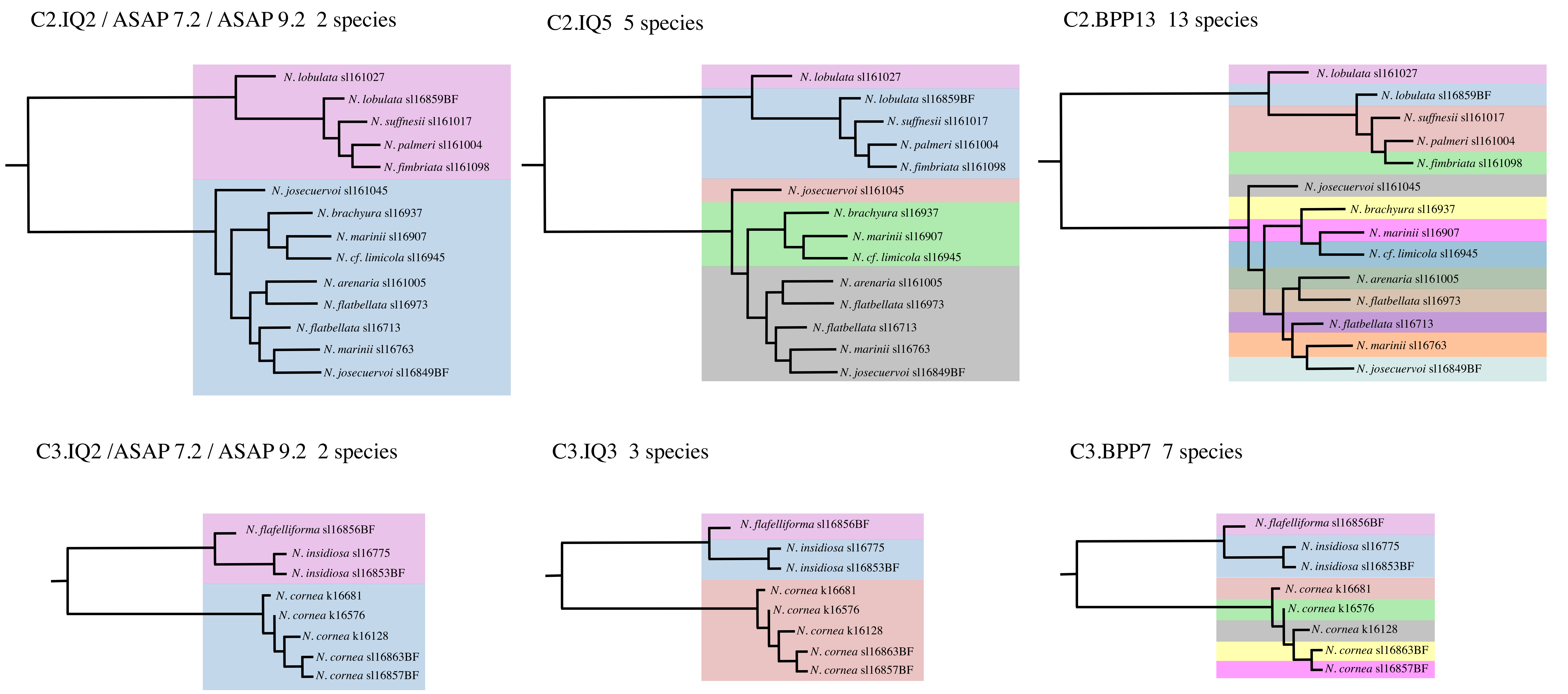
C2.IQ2 / ASAP 7.2 / ASAP 9.2 2 species
N. lobulata sl161027
N. lobulata sl16859BF
N. suffnesii sl161017
N. palmeri sl161004
N. fimbriata sl161098
N. josecuervoi sl161045
N. brachyura sl16937
N. marinii sl16907
N. cf. limicola sl16945
N. arenaria sl161005
N. flatbellata sl16973
N. flatbellata sl16713
N. marinii sl16763
N. josecuervoi sl16849BF
C2.IQ5 5 species
N. lobulata sl161027
N. lobulata sl16859BF
N. suffnesii sl161017
N. palmeri sl161004
N. fimbriata sl161098
N. josecuervoi sl161045
N. brachyura sl16937
N. marinii sl16907
N. cf. limicola sl16945
N. arenaria sl161005
N. flatbellata sl16973
N. flatbellata sl16713
N. marinii sl16763
N. josecuervoi sl16849BF
C2.BPP13 13 species
N. lobulata sl161027
N. lobulata sl16859BF
N. suffnesii sl161017
N. palmeri sl161004
N. fimbriata sl161098
N. josecuervoi sl161045
N. brachyura sl16937
N. marinii sl16907
N. cf. limicola sl16945
N. arenaria sl161005
N. flatbellata sl16973
N. flatbellata sl16713
N. marinii sl16763
N. josecuervoi sl16849BF
C3.IQ2 /ASAP 7.2 / ASAP 9.2 2 species
N. flafelliforma sl16856BF
N. insidiosa sl16775
N. insidiosa sl16853BF
N. cornea k16681
N. cornea k16576
N. cornea k16128
N. cornea sl16863BF
N. cornea sl16857BF
C3.IQ3 3 species
N. flafelliforma sl16856BF
N. insidiosa sl16775
N. insidiosa sl16853BF
N. cornea k16681
N. cornea k16576
N. cornea k16128
N. cornea sl16863BF
N. cornea sl16857BF
C3.BPP7 7 species
N. flafelliforma sl16856BF
N. insidiosa sl16775
N. insidiosa sl16853BF
N. cornea k16681
N. cornea k16576
N. cornea k16128
N. cornea sl16863BF
N. cornea sl16857BF

Clade 1

Model ID	Species	MLE_1	MLE_2	MLE_3	MLE_4	MLE	Rank	BF	ESS Range
C1.IQ3 / ASAP 7.3	3	-18145.701	-18143.544	-18147.073	-18139.088	-18143.851	5	-	4-735
C1.IQ5	5	-14419.502	-14420.782	-14429.176	-14429.811	-14424.818	3	-7438.0671	579-739
C1.IQ9	9	-13913.546	-13908.041	-13896.946	-13933.454	-13912.997	2	-8461.7095	5-115
C1.ASAP 9.5	5	-17218.573	-17218.573	-17219.231	-17232.771	-17222.287	4	-1843.1283	17-124
C1. BPP16	16	-13843.047				-13843.047	1	-8601.6094	2-154

Clade 2

Model ID	Species	MLE_1	MLE_2	MLE_3	MLE_4	MLE	Rank	BF	
C2.IQ2 / ASAP 7.2 / ASAP 9.2	2	-8857.4025	-8857.3244	-8857.5568	-8857.5798	-8857.4659	3	-	210-386
C2.IQ5	5	-8168.3869	-8191.362	-8163.7763	-8165.8956	-8172.3552	1	-1370.2214	5-159
C2.BPP13	13	-8354.8764	-8355.1223	-8354.409	-8355.5082	-8354.979	2	-1004.9739	653-865

[illegible]lade 3

Model ID	Species	MLE_1	MLE_2	MLE_3	MLE_4	MLE	Rank	BF	
C3.IQ2 / ASAP 7.2 / ASAP 9.2	2	-3934.111	-3934.1509	-3934.0632	-3934.1284	-3934.1134	3	-	818-901
C3.IQ3	3	-3829.7835	-3830.4864	-3830.6857	-3829.7738	-3830.1824	2	-207.86207	765-901
C3.BPP7	7	-3812.8804	-3819.7294	-3812.7437	-3816.8341	-3815.5469	1	-237.13291	478-641
