# Supplementary figures and images for: Species boundaries in the messy middle—A genome‐scale validation of species delimitation in a recently diverged lineage of coastal fog desert lichen fungi
Source: Ecol Evol. 2021 Dec 19;11(24):18615–32. doi: 10.1002/ece3.8467 (PMC8717302; doi:10.1002/ece3.8467)

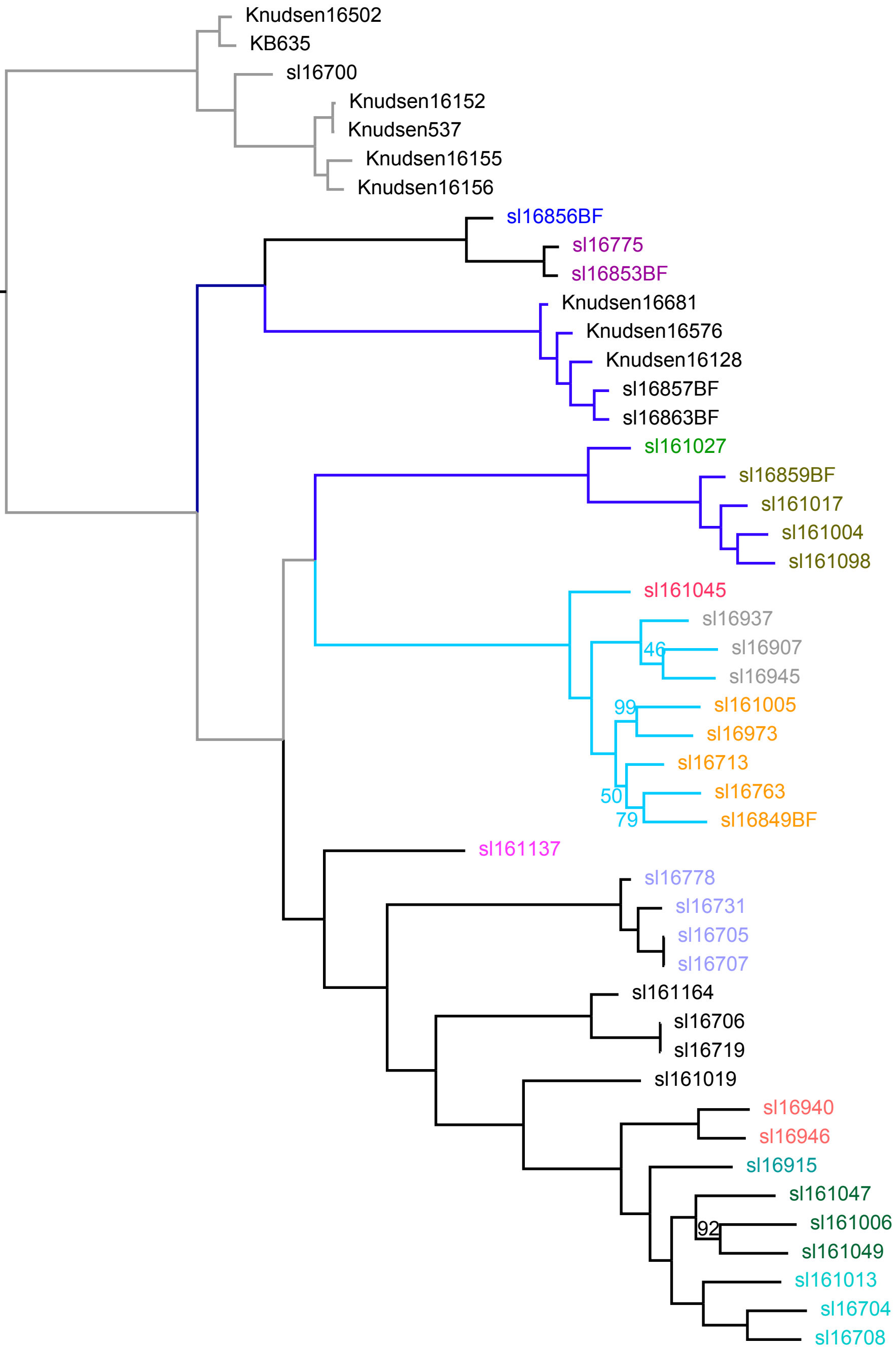

0.003

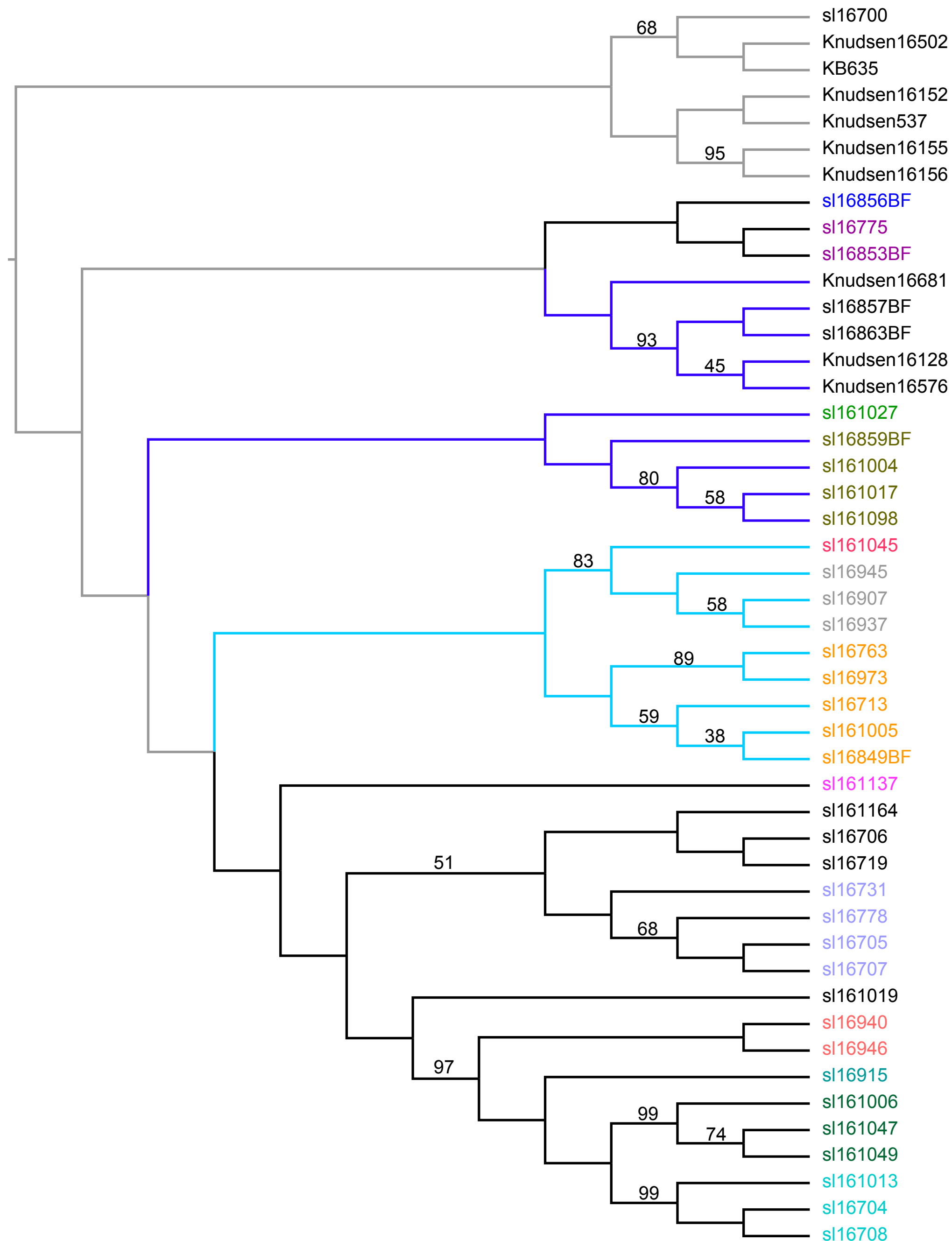

Supplement: Supplementary file 5 — Supplementary_ S5_topologies_IQtree_svdquartets [file ECE3-11-18615-s004.pdf]

# TreeMix Topology

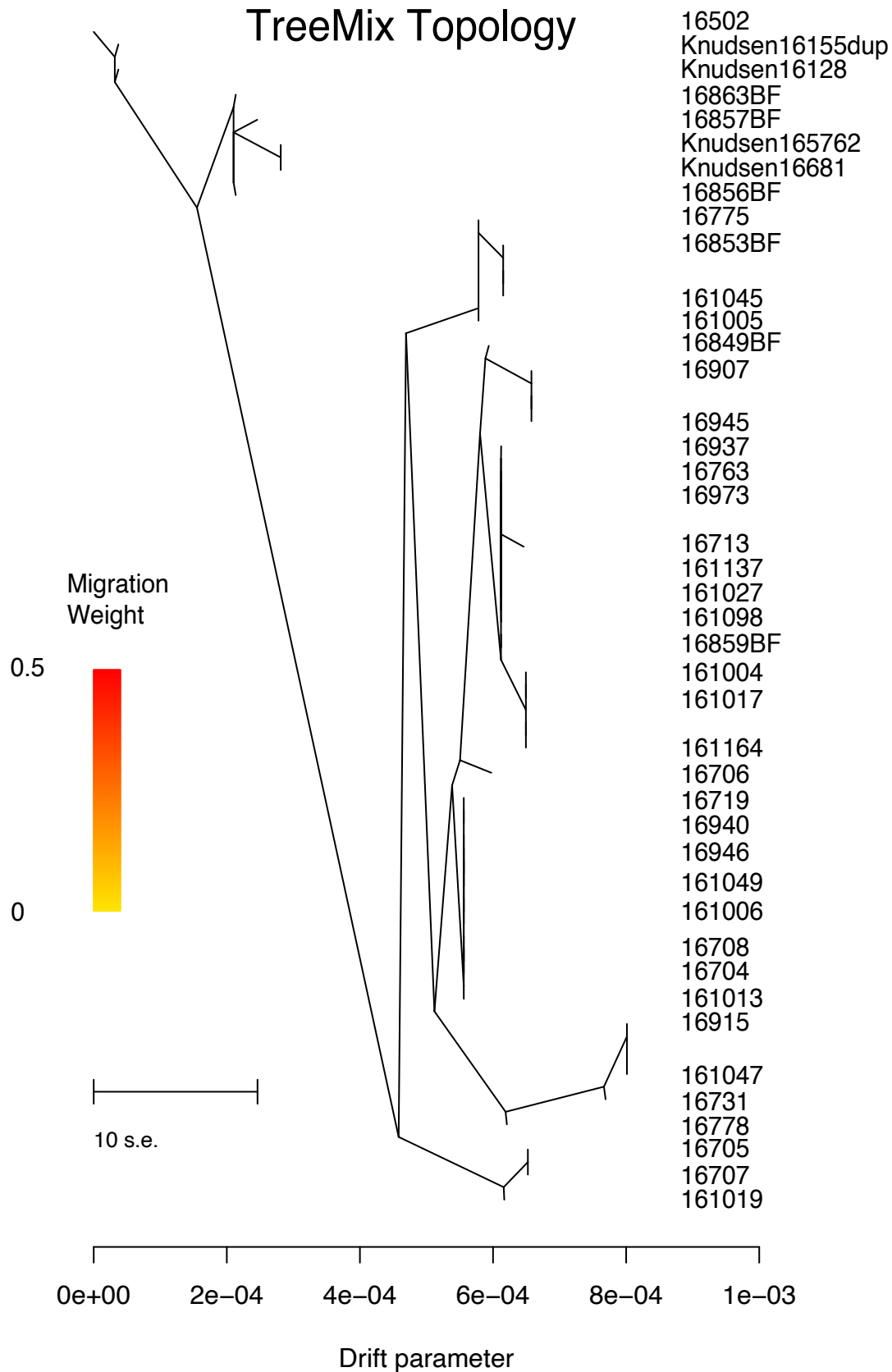

Supplement: Supplementary file 8 — Supplementary_ S8_treemix [file ECE3-11-18615-s005.pdf]
