# Supplementary material for: Species boundaries in the messy middle—A genome‐scale validation of species delimitation in a recently diverged lineage of coastal fog desert lichen fungi
Source: Ecol Evol. 2021 Dec 19;11(24):18615–32. doi: 10.1002/ece3.8467 (PMC8717302; doi:10.1002/ece3.8467)
Supplement: Supplementary file 7 — Supplementary Material [file ECE3-11-18615-s001.docx]

**Dryad file T2.** Species delimitation results from the ‘A10’ analysis in BPP and theta and tau parameter estimates from the ‘A00’ analysis in BPP under different size loci subsets and resulting average *gdi* values. The most likely delimitation model is displayed in underscore with the range of all estimations assigned some posterior probability in brackets. The fraction is the averaged posterior probability of the most likely delimitation model across the entire data subset. Theta is averaged for each individual species’ group in the 17 species models across the entire data subset, and tau here is given for the most recent common ancestor for all species (Ancestral node).

| RADseq subset | Subset criterion | Most likely delimitation model | *θ* | Standard Deviation | *τ* | Average GDI |
| --- | --- | --- | --- | --- | --- | --- |
| 10 | most informative | 1 (1-17); 0.17 | 3.73E-05 | 5.73E-07 | 9.67E-06 | 0.344 |
| 163 | ≥ 20 variable sites/locus | 15 (3-17); 0.28 | 8.85E-05 | 1.66E-05 | 5.30E-05 | 0.622 |
| 500 | most informative | 1 (1-17); 0.33 | 5.96E-05 | 1.42E-05 | 1.14E-04 | 0.89 |
| 100 | random | 16 (3-17); 0.21 | 3.31E-05 | 6.78E-06 | 6.17E-05 | 0.916 |
| 500 | random | 14 (1-17); 0.25 | 2.42E-05 | 5.93E-06 | 7.41E-05 | 0.955 |
| 1000 | random | 15 (4-17); 0.31 | 2.06E-05 | 5.62E-06 | 6.58E-05 | 0.959 |
| 5000 | random | 15 (10-17); 0.50 | - | - | - | - |
| 5649 | highly informative | - | 1.34E-05 | 6.21E-06 | 7.58E-05 | 0.976 |
